# Historic air pollution exposure and long-term mortality risks in England and Wales: prospective longitudinal cohort study

**DOI:** 10.1136/thoraxjnl-2015-207111

**Published:** 2016-02-09

**Authors:** Anna Hansell, Rebecca E Ghosh, Marta Blangiardo, Chloe Perkins, Danielle Vienneau, Kayoung Goffe, David Briggs, John Gulliver

**Affiliations:** 1 UK Small Area Health Statistics Unit, MRC-PHE Centre for Environment and Health, School of Public Health, Imperial College London, London, UK; 2 Imperial College Healthcare NHS Trust, London, UK; 3 Swiss Tropical and Public Health Institute, Basel, Switzerland; 4 University of Basel, Basel, Switzerland; 5 Department of Epidemiology and Biostatistics, Imperial College, London, UK

## Abstract

**Introduction:**

Long-term air pollution exposure contributes to mortality but there are few studies examining effects of very long-term (>25 years) exposures.

**Methods:**

This study investigated modelled air pollution concentrations at residence for 1971, 1981, 1991 (black smoke (BS) and SO_2_) and 2001 (PM_10_) in relation to mortality up to 2009 in 367 658 members of the longitudinal survey, a 1% sample of the English Census. Outcomes were all-cause (excluding accidents), cardiovascular (CV) and respiratory mortality.

**Results:**

BS and SO_2_ exposures remained associated with mortality decades after exposure—BS exposure in 1971 was significantly associated with all-cause (OR 1.02 (95% CI 1.01 to 1.04)) and respiratory (OR 1.05 (95% CI 1.01 to 1.09)) mortality in 2002–2009 (ORs expressed per 10 μg/m^3^). Largest effect sizes were seen for more recent exposures and for respiratory disease. PM_10_ exposure in 2001 was associated with all outcomes in 2002–2009 with stronger associations for respiratory (OR 1.22 (95% CI 1.04 to 1.44)) than CV mortality (OR 1.12 (95% CI 1.01 to 1.25)). Adjusting PM_10_ for past BS and SO_2_ exposures in 1971, 1981 and 1991 reduced the all-cause OR to 1.16 (95% CI 1.07 to 1.26) while CV and respiratory associations lost significance, suggesting confounding by past air pollution exposure, but there was no evidence for effect modification. Limitations include limited information on confounding by smoking and exposure misclassification of historic exposures.

**Conclusions:**

This large national study suggests that air pollution exposure has long-term effects on mortality that persist decades after exposure, and that historic air pollution exposures influence current estimates of associations between air pollution and mortality.

**Key messages**

**What is the key question?**

- What is the impact of very long-term (>30 years) air pollution exposure on mortality?

**What is the bottom line?**

- Historic air pollution exposure has long-term effects on mortality that persist over 30 years after exposure and these potentially also influence current estimates of associations between air pollution and mortality.

**Why read on?**

- This is one of the longest running studies to look at health effects of air pollution, using air pollution estimates independently assessed at multiple time points using contemporaneous monitoring data in a large cohort followed for 38 years.

## Introduction

While the impact of air pollution on mortality in the short term (days) and medium term (<10 years) is now well established, there are relatively few studies assessing the long-term (>10 years) impact of air pollution^1–10^ with even fewer assessing the very long term (25+ years).^2^ ^3^ ^8–10^ Only a small number of these^4–6^ had exposure data at more than one time point.

Like many other developed countries the UK experienced high levels of air pollution in the past, including the infamous London smog episode of December 1952,^11^ since when air pollution levels have fallen to much lower levels. Changes in air pollution concentrations in the UK are well documented as, uniquely, the UK had a comprehensive national air quality monitoring network running from the 1950s to the 1990s measuring black smoke (BS) and sulfur dioxide (SO_2_) arising from domestic and industrial coal and fossil fuel combustion, then major sources of emissions. Thereafter, networks switched to monitor nitrogen dioxide (NO_2_) (from the early 1990s) and particulate matter with a diameter of 10µm or less (PM_10_) (from the mid-1990s), as transport emissions became the largest source of air pollution.^12^ ^13^ The present study uses a very large nationally representative British cohort to consider impact of air pollution over 38 years of follow-up. Three a priori hypotheses were investigated:

1. Historic air pollution (ie, of several decades previously) is associated with later mortality risk.
2. The mortality risks associated with a given exposure decrease over subsequent decades.
3. Air pollution exposures in previous decades interact with recent exposures to affect mortality risk.

## Methods

This investigation used a long-running census-based study, the Office for National Statistics (ONS) Longitudinal Study, which contains linked census and life events data on a representative 1% sample of the population of England and Wales. The initial sample was drawn from the 1971 census.^14^ ^15^

**Figure 1:**
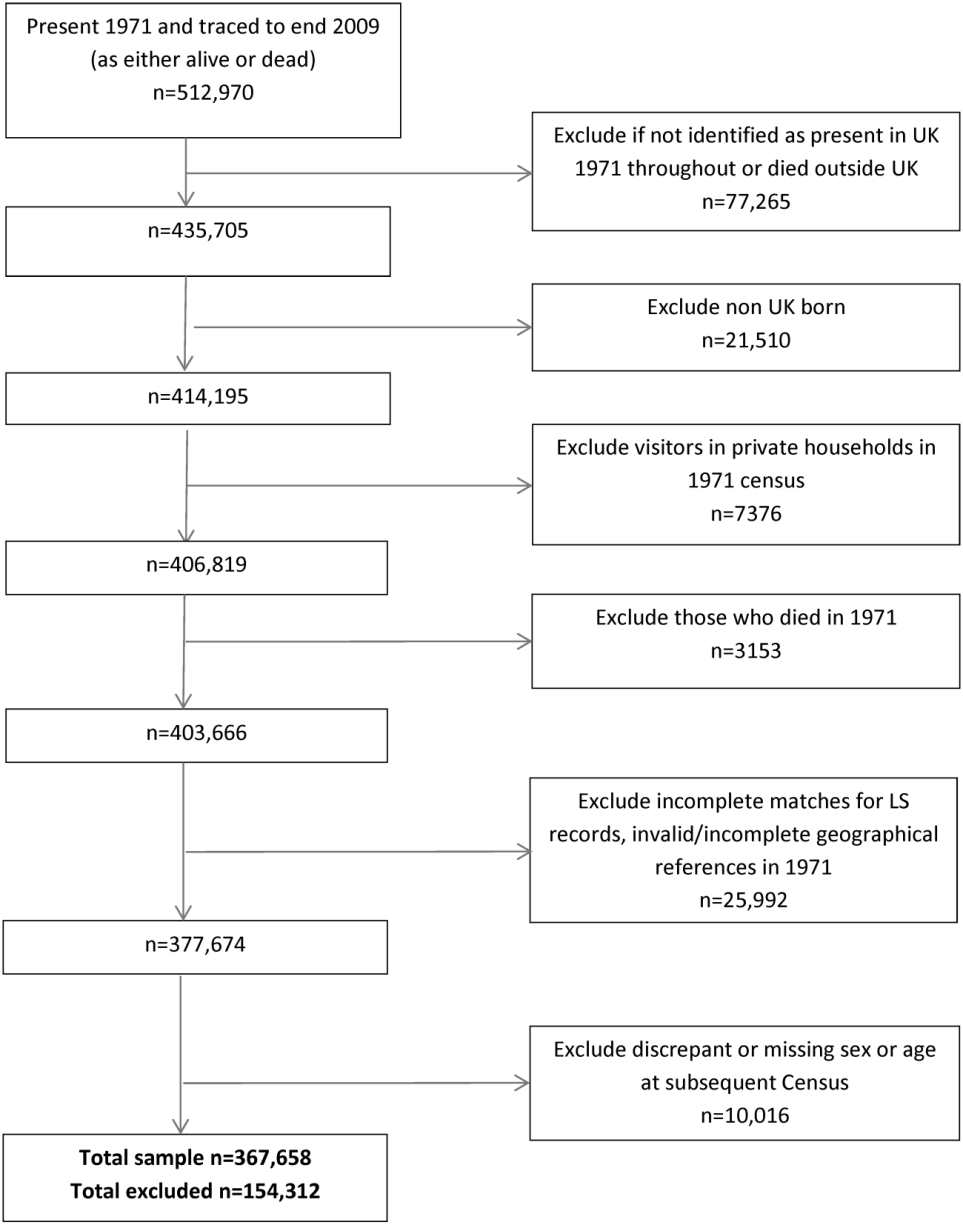
Identification of longitudinal survey (LS) participants. Source: Office for National Statistics Longitudinal Study (authors’ own work).

For this investigation, the study was restricted to members of the cohort of all ages present at the 1971 census, who were either present at each subsequent census (1981, 1991 and 2001) and either traced up to 2009 or had died, who were not identified through general practice (GP) registration as having left the country. Exclusions (figure 1) were made for data inaccuracies, those who died in 1971 and those not UK born (who may have had different previous air pollution exposures). By constructing a closed cohort, we were able to estimate air pollution exposures across the entire period of their life 1971–2009 for each individual.

### Air pollution exposures in 1971, 1981, 1991 and 2001

Land use regression techniques were used to model BS and SO_2_ annual concentrations in 1971, 1981 and 1991 at 1 km grids. Models for BS and SO_2_ have been described in detail previously^12^ but were developed with a range of variables including information on land cover, major and minor roads, and X–Y coordinates of each monitoring site. Models were developed against concentration data from national monitoring station sites where operational days in the year exceeded 75%, which involved a total of 966 sites for BS and 825 sites for SO_2_. Model building used 80% of network sites; the remaining independent, randomly stratified 20% sample was retained for model validation. The validation statistics from the independent subset gave r^2^ values for BS of 0.41, 0.38 and 0.34 for 1971, 1981 and 1991, respectively, and for SO_2_ of 0.57, 0.26 and 0.31. Values of mean (fractional) bias were low in all years (ie, <−0.1), which suggests that predicted values were within 20% of observed monitored concentrations. Land use regression techniques were used to model PM_10_ at 100 m grids in 2001.^16^ Leave-one-out validation statistics for PM_10_ in 2001 gave r^2^ value of 0.37.

### Integration of air pollution and confounder data into the longitudinal survey

Air pollution exposure estimates were produced for UK grids and wards (ONS small area geographical units) and Centre for Longitudinal Study Information & User Support (CeLSIUS) staff matched these to individuals; precise geolocation of longitudinal survey (LS) participants is not made available to researchers. For 1971, individuals were assigned the annual average BS and SO_2_ concentration of the 1 km grid in which their residence was located. For other years (1981, 1991 and 2001) the pollutant surfaces (ie, regular grids) were intersected with ward boundaries and area-weighting was used to calculate the average values of each pollutant within each ward.

Information on smoking was not available at individual level in the cohort, so smoothed district-level lung cancer mortality relative risk 2002–2009 (International Classification of Diseases (ICD)-10 codes C33-C34) as a proxy measure for cumulative smoking over the past 20+ years^17^ were used. Lung cancer risks using Bayesian smoothing methods were calculated from ONS data held by the Small Area Health Statistics Unit.

### Statistical analysis

Statistical analyses of individual-level data were conducted in person at the ONS offices using Stata V.11 (Stata, College Station, Texas, USA). Descriptive analyses were conducted for all variables. Logistic regression analyses (died/survived) were used to investigate associations between BS and/or SO_2_ exposure in 1971, 1981 and 1991 and PM_10_ in 2001 and risk of death in 1972–1981, 1982–1991, 1992–2001 and 2002–2009, respectively. Mortality outcomes were all-cause excluding accidents; cardiovascular (CV) and respiratory mortality and by constituent subgroups of coronary heart disease (CHD), stroke, respiratory infections, COPD and lung cancer. For ICD codes used, see the online supplementary appendix A table A1. We conducted analyses by decade to align with census years, the periodicity of which reflects marked changes in air pollution sources in the UK, from predominantly fossil fuel burning in the 1970s to domination by traffic-based sources with increasing contribution from diesel engines by 2001.

Adjustments were made: (i) for age and sex; (ii) additionally for social class of individual (Registrar General occupation) and area (quintiles of Carstairs deprivation index), population density (not used in any of the exposure models) and geographical region (in 1971). Sensitivity analyses were conducted adjusting for the smoking proxy (lung cancer risks), restricting analyses to non-movers in the 5 years prior to the 1971 census and adjusting for exposures in other years. All air pollution variables, population density and age variables were centred prior to regression analyses.

To evaluate whether past BS or SO_2_ exposure modified the effect of PM_10_ on mortality in 2002–2009 we introduced interaction terms in statistical models between tertiles of BS/SO_2_ and PM_10_ and examined risks by exposure tertiles. As SO_2_ was highly correlated with the same-year BS, we did not conduct two pollutant analyses. Finally, through a piecewise linear model for the tertiles of BS/SO_2_ at 1971 and the three main outcomes (all-cause mortality, all respiratory mortality and all CV mortality) we visually assessed the presence of a concentration–response.

## Results

### Descriptive analyses

The analyses included 367 658 individuals followed from 1971 to 2009 with non-missing data (figure 1), comprising 71.67% of the initial cohort. The main reasons for exclusion were emigration (n=77 265) and missing or incomplete data (n=25 992). Those excluded from the analysis were significantly (p<0.001) younger in 1971 than those included (mean age 25 vs 38 years), more likely to be male (54% vs 48%), more likely to have lived in most deprived areas in 1971 (23% vs 20%) and to have moved between 1966 and 1971 (59% vs 39%) (see online supplementary appendix A table A2).

Median air pollution exposures to BS and SO_2_ were twofold to threefold higher in 1971 compared with 1991. Ranges (10th–90th centiles) for BS were 18.5–70.5 μg/m^3^ in 1971 and 3–19 μg/m^3^ in 1991 (table 1).

**Table 1:** Descriptive analyses of the Longitudinal Study

	Mean (SD)	Median	10th centile	90th centile
Air pollution exposure (μg/m^3^)				
BS 1971	42.7 (20.4)	41	18.5	70.5
SO_2_ 1971	85.2 (36.8)	77	44.5	137
BS 1981	16.2 (5.2)	16	8.5	25
SO_2_ 1981	43.1 (12.1)	41.5	25.5	66
BS 1991	11.8 (4.7)	12	3	19
SO_2_ 1991	29.6 (6.5)	29.5	19	40.5
PM_10_ 2001*	20.7 (2.5)	20	18	24
Age in years				
Age in 1971	38 (22.9)	30	7	68
Age in 1981	43.3 (20.8)	42	13	77
Age in 1991	48.9 (18.6)	47	22	80
Age in 2001	54.3 (16)	53	32	82
Population density (per km) 1971	3486.2 (3075.1)	2915.6	206.4	7187.6
**Number of deaths by year**	**1972–1981**	**1982–1991**	**1992–2001**	**2002–2009**
All-cause excluding accidents	48, 834	47 775	45 736	31 744
Cardiovascular (CV)	26 140	23 923	20 054	11 876
Cardiovascular mortality: CHD	14 050	13 461	6,219	5613
Cardiovascular mortality: Stroke	6401	6189	4803	810
Respiratory	6959	5300	7302	4598
Respiratory mortality: Respiratory Infections	4219	2364	4272	2109
Respiratory mortality: COPD	2259	2413	2319	1644
Lung cancer	3185	3154	2731	1920

Gender	Number	Per cent
Males	175 532	47.7
Females	192 126	52.3
Non-movers (1966–1971)	223 055	60.7
Region 1971		
Northern	26 243	7.1
Yorkshire/Humber	39 853	10.8
North West	52 114	14.2
East Midlands	29 582	8.1
West Midlands	34 535	9.4
East Anglia	13 602	3.7
South East	67 538	18.4
South West	31 726	8.6
Wales	22 155	6.0
Inner London	17 863	4.9
Outer London	32 447	8.8
Individual-level social class 1971 (Registrar General occupational categories)		
I Professional	11 381	3.1
II Intermediate	49 427	13.5
III(N) Skilled non-manual	50 789	13.8
III(M) Skilled manual	90 082	24.5
IV Semi-skilled manual	53 415	14.5
V Unskilled manual	20 220	5.5
Armed forces and inadequately described	25 860	7.0
Other economically inactive/student/childcare/retired/permanently sick	66 260	18.0
Area-level deprivation 1971 (Carstairs quintile)		
1 (least deprived)	69 608	19.0
2	74 140	20.2
3	75 428	20.6
4	75 511	20.6
5 (most deprived)	72 202	19.7

*Given the finer 100 m resolution for PM_10_, only integer values of PM_10_ were permitted by ONS for analysis because of concerns that more decimal places might allow identifiability in the linked dataset. Source: ONS Longitudinal Study (authors’ own work).

BS, black smoke; CHD, coronary heart disease; ONS, Office for National Statistics.

The mean PM_10_ exposure in 2001 was 20.7 μg/m^3^ (10–90th centile 18–24). The highest exposures were seen in urban metropolitan areas: BS was highest in the northern regions of England and Wales, and highest SO_2_ and PM_10_ exposures were seen in London (see online supplementary appendix A table A3). All exposures decreased with increasing individual-level social class and increased with increased deprivation of area of residence (see online supplementary appendix A table A3).

PM_10_ exposure in 2001 was weakly correlated with BS and SO_2_ in earlier years (all r <0.45) (table 2).

**Table 2:** Correlations between participant air pollution estimates and potential confounders

	BS 1971	SO_2_ 1971	BS 1981	SO_2_ 1981	BS 1991	SO_2_ 1991	PM_10_ 2001	Age	Population density	RR lung cancer
BS 1971 n=367 658	1.000									
SO_2_ 1971 n=367 658	0.730	1.000								
BS 1981 n=305 471	0.696	0.531	1.000							
SO_2_ 1981 n=305 471	0.519	0.720	0.762	1.000						
BS 1991 n=259 649	0.651	0.405	0.769	0.538	1.000					
SO_2_ 1991 n=259 649	0.645	0.452	0.750	0.600	0.866	1.000				
PM_10_ 2001 n=221 148	0.195	0.411	0.200	0.413	0.077	0.190	1.000			
Age n=367 658	−0.002	0.012	−0.020	−0.005	−0.035	−0.034	−0.014	1.000		
Population density n=367 658	0.441	0.689	0.252	0.438	0.044	0.107	0.380	0.031	1.000	
RR lung cancer n=221 148	0.059	−0.162	0.010	−0.202	0.007	0.047	−0.191	0.004	−0.150	1.000

Population Density—population density 1 km grid. Source: Office for National Statistics Longitudinal Study (authors’ own work).

BS, black smoke.

Within-year BS and SO_2_ exposures were highly correlated (r>0.7). Correlations were also moderate to high (r∼0.6–0.7) for BS exposures between years, but there was a greater range for SO_2_ (r∼0.45–0.7).

### BS exposures in 1971–1991

**Figure 2:**
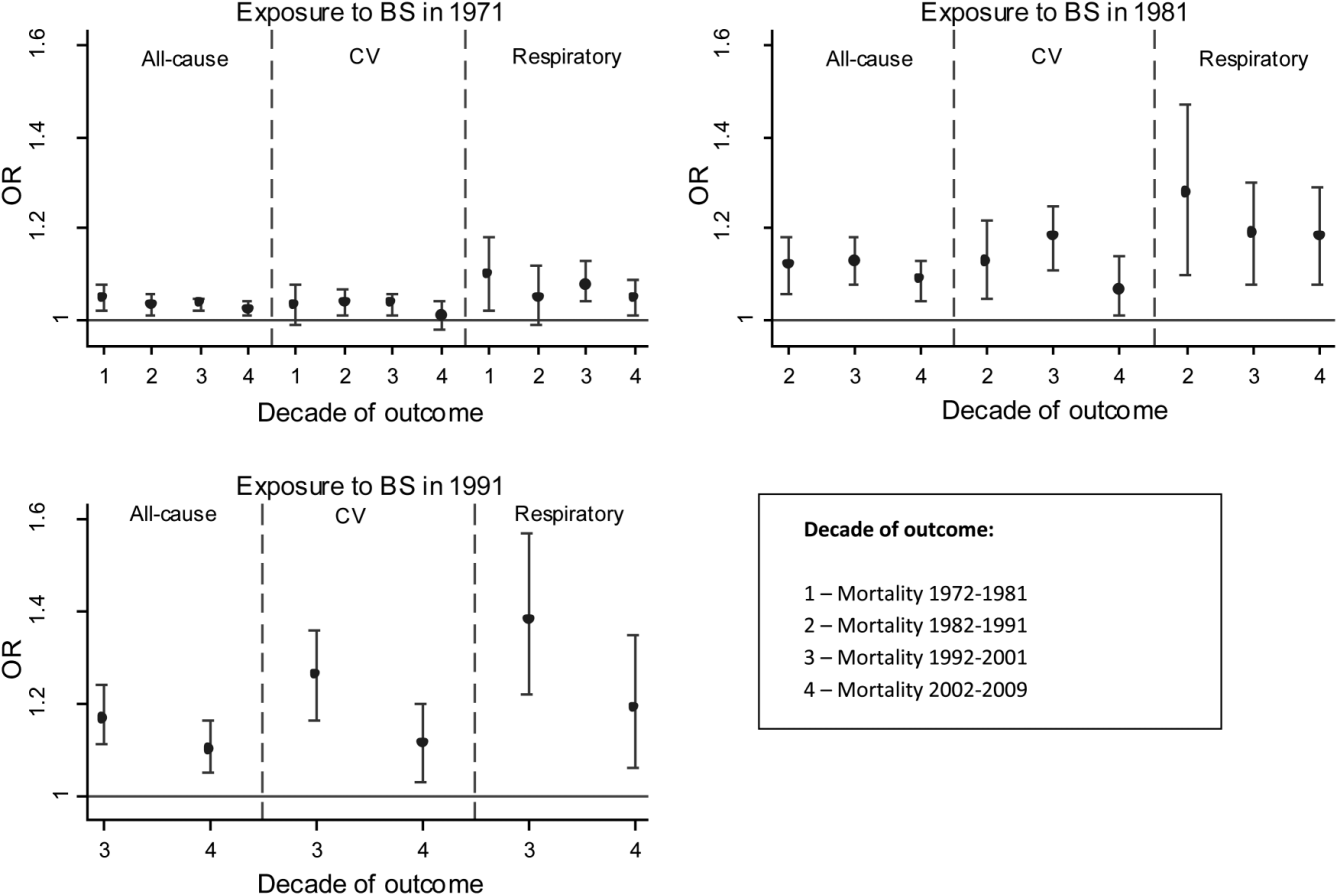
ORs (95% CI) per 10 μg/m^3^ for black smoke (BS) exposure in 1971, 1981 and 1991 and mortality in subsequent decades. Adjusted for age and sex, social class of individual and area, population density and geographical region. Source: Office for National Statistics Longitudinal Study (authors’ own work).

There were statistically significant associations between BS exposure in 1971 and all-cause mortality in all subsequent decades through to 2002–2009 (figure 2 and table 3); CV and respiratory mortality showed similar patterns as all-cause mortality.

**Table 3:** Logistic regression ORs (95% CI) per 10 μg/m^3^ for black smoke exposure in 1971 and mortality in subsequent decades

Decade of outcome	(i) Unadjusted (age and sex only)	(ii) Adjusted (age, sex, social class, area-level deprivation, region, population density)
All-cause mortality excluding accidents		
1972–2009	1.07 (1.07 to 1.08)	1.03 (1.02 to 1.05)
1972–1981	1.10 (1.08 to 1.11)	1.05 (1.02 to 1.08)
1982–1991	1.09 (1.08 to 1.10)	1.03 (1.01 to 1.06)
1992–2001	1.07 (1.07 to 1.08)	1.04 (1.02 to 1.05)
2002–2009	1.05 (1.05 to 1.06)	1.02 (1.01 to 1.04)
Cardiovascular mortality		
1972–2009	1.08 (1.07 to 1.09)	1.03 (1.01 to 1.05)
1972–1981	1.11 (1.09 to 1.13)	1.03 (0.99 to 1.08)
1982–1991	1.10 (1.09 to 1.12)	1.04 (1.01 to 1.07)
1992–2001	1.08 (1.07 to 1.09)	1.04 (1.01 to 1.06)
2002–2009	1.05 (1.04 to 1.06)	1.01 (0.98 to 1.04)
Respiratory mortality		
1972–2009	1.11 (1.10 to 1.12)	1.07 (1.04 to 1.10)
1972–1981	1.12 (1.09 to 1.16)	1.10 (1.02 to 1.18)
1982–1991	1.14 (1.12 to 1.17)	1.05 (0.99 to 1.12)
1992–2001	1.10 (1.08 to 1.12)	1.08 (1.04 to 1.13)
2002–2009	1.09 (1.07 to 1.11)	1.05 (1.01 to 1.09)

Source: Office for National Statistics Longitudinal Study (authors’ own work).

**Figure 3:**
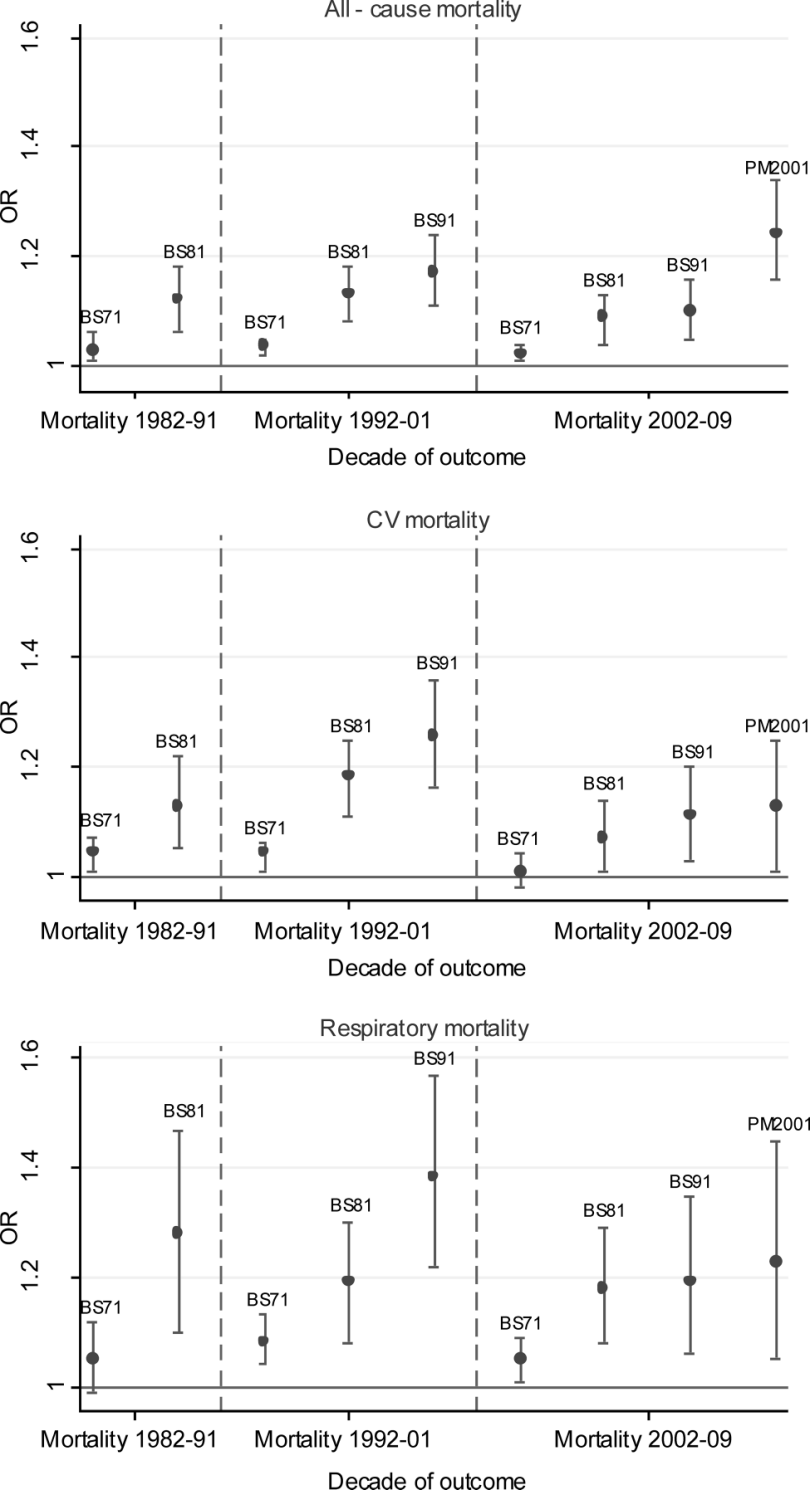
ORs (95% CI) per 10 μg/m^3^ for BS exposure in 1971, 1981 and 1991 and PM_10_ in 2001 and mortality in each subsequent decades. Adjusted for age and sex, social class of individual and area, population density and geographical region. Source: ONS Longitudinal Study (authors’ own work). CV, BS, black smoke; cardiovascular disease; ONS, Office for National Statistics.

BS exposures in 1981 and 1991 were also significantly associated with all-cause, CV and respiratory mortality in subsequent decades (see figure 2 and online supplementary appendix B table B1). Figure 3 shows stronger effects for more recent BS exposures. Largest associations were between BS exposure in 1991 and respiratory mortality in 1991–2001 with OR 1.38 (95% CI 1.22 to 1.57) (see online supplementary appendix B table B1). In the subgroup analyses (see online supplementary appendix B table B1), risks were marginally higher for CHD than stroke mortality and higher for COPD and lung cancer than for respiratory infections especially for more recent exposures. The highest risks observed were for COPD mortality. COPD mortality remained significantly associated with past exposures up to the most recent decade, while respiratory infections generally reduced in magnitude and became non-significant over time.

Adjustment for confounders slightly reduced ORs for exposures in 1971 (table 3) and 1991 (see online supplementary appendix B table B1) but showed a larger effect for BS in 1981 (see online supplementary appendix B table B1). Sensitivity analyses restricted to the non-movers made no difference in ORs. The association with respiratory mortality in 2002–2009 no longer reached statistical significance after additional adjustment for the smoking proxy in 2002–2009 but it made no difference to associations with all-cause and CV mortality (see online supplementary appendix B table B2).

**Figure 4:**
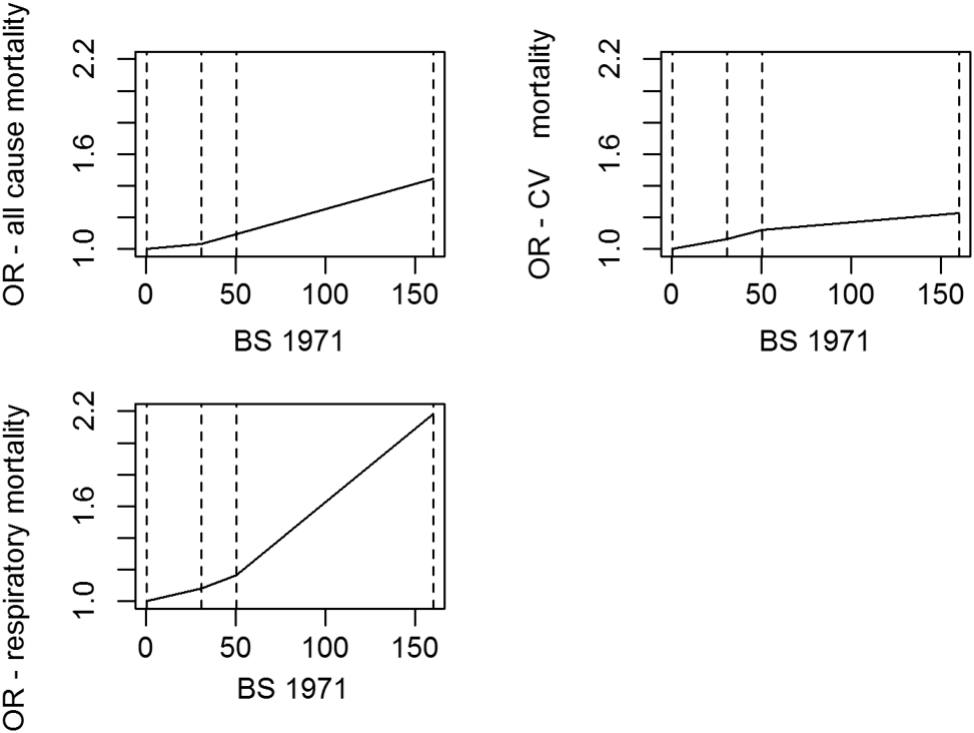
Concentration-response for tertiles of BS exposure in 1971 and subsequent mortality 1971-2009 for all-cause, CV and respiratory mortality. Adjusted for age and sex, social class of individual and area, population density and geographical region. Source: ONS Longitudinal Study (authors' own work). BS, black smoke; CV, cardiovascular disease.

Concentration–response for each tertile of BS exposure in 1971 for the three main outcomes (figure 4) showed a steeper response in the highest tertiles that was most marked for respiratory mortality. By 1981, the 90th centile value of BS exposure (25 μg/m^3^) was within the lowest tertile range of BS exposure for 1971 (1971 tertile cut points 31 and 50.5 μg/m^3^).

### SO_2_ exposures in 1971–1991

Results for SO_2_ showed very similar patterns to those for BS (see online supplementary appendix C) including for concentration–response. As for BS, there were statistically significant associations between SO_2_ exposure in 1971 and mortality in all subsequent decades through to 2002–2009 (see online supplementary appendix C figure C1 and table C1). The largest association, as for BS, was between SO_2_ exposure in 1991 and respiratory mortality in 1991–2001 with OR 1.24 (95% CI 1.15 to 1.34) (see online supplementary appendix C table C1).

### PM_10_ exposure in 2001

PM_10_ exposure in 2001 was associated with an increased risk of all-cause mortality in 2002–2009 after adjustment (OR 1.24 (95% CI 1.15 to 1.33)) (table 4), with higher ORs for respiratory and lower for CV mortality. Within the subgroup analyses, the highest risk observed was for lung cancer with OR 1.60 (1.29 to 1.99) (see online supplementary appendix B table B4).

**Table 4:** Logistic regression ORs (95% CI) per 10 μg/m^3^ for PM_10_ exposure in 2001 and mortality in 2002–2009 adjusted for past black smoke (BS) exposure

2002–2009	(i) Unadjusted (age and sex only)	(ii) Adjusted (age, sex, social class, area-level deprivation, region, population density)	Sensitivity analysis: adjusted (age, sex, social class, area-level deprivation, region, population density and lung cancer)
All-cause mortality excluding accidents			
PM_10_ 2001	1.37 (1.29 to 1.45)	1.24 (1.16 to 1.34)	1.24 (1.15 to 1.33)
PM_10_ 2001+BS 1971	1.27 (1.19 to 1.35)	1.26 (1.17 to 1.37)	1.23 (1.14 to 1.32)
PM_10_ 2001+BS 1981	1.25 (1.17 to 1.33)	1.19 (1.11 to 1.28)	1.19 (1.10 to 1.28)
PM_10_ 2001+BS 1991	1.29 (1.21 to 1.38)	1.20 (1.11 to 1.29)	1.19 (1.11 to 1.28)
PM_10_ 2001+BS all years	1.18 (1.11 to 1.26)	1.21 (1.11 to 1.31)	1.16 (1.07 to 1.25)
Cardiovascular mortality			
PM_10_ 2001	1.25 (1.14 to 1.37)	1.12 (1.01 to 1.25)	1.12 (1.01 to 1.25)
PM_10_ 2001+BS 1971	1.16 (1.06 to 1.27)	1.12 (1.01 to 1.25)	1.12 (1.00 to 1.24)
PM_10_ 2001+BS 1981	1.15 (1.03 to 1.27)	1.09 (0.98 to 1.22)	1.09 (0.97 to 1.22)
PM_10_ 2001+BS 1991	1.18 (1.07 to 1.29)	1.08 (0.97 to 1.20)	1.07 (0.96 to 1.20)
PM_10_ 2001+BS all years	1.09 (0.98 to 1.20)	1.06 (0.95 to 1.19)	1.06 (0.94 to 1.19)
Respiratory mortality			
PM_10_ 2001	1.55 (1.35 to 1.77)	1.23 (1.05 to 1.45)	1.22 (1.04 to 1.44)
PM_10_ 2001+BS 1971	1.36 (1.19 to 1.57)	1.22 (1.04 to 1.43)	1.21 (1.03 to 1.42)
PM_10_ 2001+BS 1981	1.38 (1.20 to 1.59)	1.18 (1.00 to 1.39)	1.17 (0.99 to 1.38)
PM_10_ 2001+BS 1991	1.50 (1.31 to 1.72)	1.21 (1.02 to 1.42)	1.20 (1.02 to 1.41)
PM_10_ 2001+BS all years	1.33 (1.14 to 1.54)	1.18 (0.99 to 1.40)	1.17 (0.98 to 1.39)

*Source*: Office for National Statistics Longitudinal Study (authors’ own work).

Additional adjustment for past exposures to BS in 1971, 1981 and 1991 reduced ORs for each outcome and the OR for CV mortality (CV) lost statistical significance. The impact of adjusting for past air pollution exposures was greater than that produced by adjusting for individual social class and area-level deprivation. Similar effects were seen whether past BS, SO_2_ or both were adjusted for, as expected from the high correlations between BS and SO_2_ (see online supplementary appendix B table B3).

There were no clear patterns in interaction terms between PM_10_ in 2001 in relation to mortality in 2002–2009 and past exposures in terms of tertiles of BS or SO_2_ exposure in 1971, 1981 or 1991, or always highest or always lowest tertiles of exposures. The exception to this was for CV with respect to BS in 1981 (p value for highest tertile of exposure=0.014), suggestive of lower OR for PM_10_ and CV confined to the highest tertile of BS exposure in 1981, which is an apparently protective effect.

## Discussion

This study investigated air pollution exposures in 370 000 individuals in a national census-based cohort followed for 38 years. In line with our prior hypotheses, we found that historic exposures to BS and SO_2_ were associated with increased risks of all-cause, CV and respiratory mortality in England and Wales over 30 years later, and mortality risks associated with a given exposure generally decreased over time. Subgroup analyses showed highest risks for COPD and lung cancer mortality. Adjusting for past BS or SO_2_ exposures resulted in slightly lower observed mortality associations with recent PM_10_ exposure (suggestive of confounding), but there was no clear evidence that higher air pollution exposures in earlier life resulted in greater or lesser susceptibility to PM_10_ (effect modification).

We saw highest associations with respiratory mortality, consistent with other UK-based studies investigating long-term BS exposures in the 1950s and 1960s,^18^ 1970s,^7^ 1980s and 1990s^5^ and PM_10_ in the 2000s^19^ and with a population-registry-based study in the Netherlands examining PM_10_ exposure in 2001.^20^ In contrast, studies of large American cohorts have found highest associations of particulates with CV mortality,^4^ ^6^ as did a recent study in Rome,^21^ while the large European Study of Cohorts and Air Pollution Effects (ESCAPE) analyses found associations with all-cause^9^ but not non-malignant respiratory^8^ or CV mortality.^10^ Reasons for these differences are unclear, but may include differences in death certification practices between countries.^22^ ^23^

Our effect sizes for BS were very similar to those of recently reported previous British studies^5^ ^7^ especially for more recent exposures, despite differing exposure estimation methods and study design. The only other UK study to investigate recent exposure to PM_10_ by Carey *et al*^19^ reported a lower effect size of HR 1.07 (0.99 to 1.16) for all-cause mortality in 2003–2007, compared with OR 1.24 (1.16 to 1.34) in this study. Effect estimates are not incompatible but non-differential exposure misclassification in the Carey study (where PM_10_ concentration estimates were on 1 km grids, compared with 100 m grids in this study) may have biased estimates towards the null, and alternatively the lower estimates in the Carey study may relate to better control for individual-level confounders. Our effect size for PM_10_ exposure in 2001 and lung cancer mortality in 2002–2009, OR 1.60 (1.29 to 1.99), is larger than the combined estimate from 14 cohorts in the ESCAPE study (HR 1.22 (1.03 to 1.45)),^24^ although our CIs overlap. The ESCAPE study was able to adjust for smoking, which we were not able to do for this outcome as our smoking proxy was lung cancer. Some earlier UK studies of air pollution in the 1970s,^1^ ^25^ including one using the LS,^1^ did not find associations between mortality and particulate air pollution. This may relate to previous less accurate air pollution assessment based on nearest monitoring station.

Few studies have examined long-term effects of SO_2_ exposures. Studies conducted in the UK^5^ ^19^ ^25^ and the American Cancer Society in the USA^26^ have generally found statistically significant associations of mortality with SO_2_, while studies in other parts of Europe^2^ ^3^ ^27^ have not. In the present study, BS and SO_2_ levels were highly correlated (both originated from fossil fuel combustion), so it is difficult to clearly attribute mortality effects to one pollutant.

### Was apparent persistence of air pollution mortality risk due to highly correlated exposures?

Our results showed continued effects air pollution from 1971 on mortality in subsequent decades up to 2002–2009, suggesting long-term persistence of risk. Since 1966 (5 years prior to the start of the study in 1971) and 2001, 72% of individuals had moved at least once, so results are not merely a function of living in the same place. Air pollution exposures were assigned to an individual's ward of residence (or 1 km grid of residence) at each census year. BS and SO_2_ air pollution exposures were moderately correlated between decades (r∼0.6–0.7), so an alternative explanation for the apparent persistence of risks to 2002–2009 is that exposure in 1971 may have been acting as a proxy for more recent exposures. However, while adjustment for BS in subsequent years (1981 and 1991) did reduce effect estimates, those for all-cause and respiratory mortality remained statistically significant (see online supplementary appendix B table B2). It is also possible that 1971 levels of exposure may correlate highly with and be a proxy for earlier life exposures when levels were much higher.

### Mortality risks associated with a given exposure decrease over time

Mortality risks were generally highest in decades immediately following exposure. This is consistent with a previous UK study finding that in the last 4 years BS and SO_2_ exposure gave higher risks of respiratory mortality than exposures 12–16 years prior.^5^ It is also consistent with the Harvard Six Cities follow-up study,^4^ which found similar risks for annual compared with mean air pollution over the study period (1980–1998), suggesting that air pollution during the last year may be important. The follow-up of the American Cancer Society study^6^ did not find clear differences in risks for average PM_2.5_ and SO_2_ concentrations 1–5, 6–10 and 11–15 years before death, but high correlations between the time periods may have reduced the ability to detect differences.

Increased risks per unit pollutant were observed for more recent exposures even though air pollution levels fell markedly (fourfold lower mean BS concentrations between 1971 and 1991). This might imply a steeper concentration–response curve at lower exposures,^28^ although this was not the case for BS exposure in 1971 where a steeper concentration–response curve was seen in higher exposures (figure 4); or be due to more accurate exposure estimates for more recent periods. However, the latter seems unlikely because exposure models performed better for earlier periods. Alternatively, changes in air pollution sources over time, with reductions in industry and household emissions and increases from road traffic,^12^ ^13^ may have led to changes in toxicity. Due to qualitative changes in particulate composition over time, we did not adopt a conversion factor between PM_10_ and BS. However, the two measures are moderately highly (r=0.5–0.8) correlated^29^ and a number of previous studies have found associations between BS and CV^30^ and respiratory mortality^31^ with comparable effect sizes expressed per IQR of exposure.^29^ The larger size of the PM_10_ associations relative to the BS per unit mass in the present study may suggest greater toxicity of recent particles or changes in population susceptibility. A ‘harvesting’ effect of sensitive individuals in our closed cohort might at least partly account for waning effects of air pollution over time, but would be inconsistent with increased risks for more recent exposures, unless sensitivity also increased over time.

### Interaction of past with recent air pollution exposures

We did not find evidence for effect modification by past exposures to BS and SO_2_, or put another way, we did not find that higher exposures in earlier life has a multiplicative effect on mortality risk associated with more recent PM_10_ exposure. Our results did suggest that the relationship between mortality and PM_10_ exposure was confounded by past exposure (ie, that past air pollution exposures are independently associated with both PM_10_ exposure and with mortality outcomes, suggesting that not accounting for past exposures will affect observed risk estimates for more recent exposures), although the overall impact of this was small. This is one of the first studies directly to investigate this question. This confounding is unlikely to be solely due to correlation between the exposures as PM_10_ exposure in 2001 was not strongly correlated with BS and SO_2_ in earlier years (all r<0.45).

### Exposure assessment

Previous studies investigating very long-term air pollution exposures have had limited historical exposure information. ESCAPE studies^8–10^ relied on back-extrapolation from modelled exposures in 2008–2011, studies in Stockholm used emissions data^3^ ^32^ to estimate historic SO_2_ exposure without independent measured concentrations to evaluate models, while other studies have used data from the nearest monitoring station.^2^ ^5^ ^26^ We used land-use regression models, validated against contemporaneous monitored concentrations. Model performance was moderate (r^2^ 0.3–0.5) with weakest performance for SO_2_ in 1981 (r^2^=0.26) and best performance for SO_2_ in 1971 (r^2^=0.57),^12^ but the lower r^2^ values in later years may be partly related to lower variability in concentrations rather than model performance.

We used air pollution estimates to the highest spatial resolution available, which were on 1 km (BS and SO_2_) and 100 m (PM_10_) exposure grids. This may have contributed to some exposure misclassification, though this is likely to be non-differential and result in bias towards the null. Because of limited access to location of individuals in the LS cohort, we were unable to adjust for spatial autocorrelation, but other studies suggest that impact of this is small.^6^ ^19^

### Other study limitations

While the cohort was originally a population-based sample, losses to follow-up occurred that were higher in those living in more deprived areas. We consider that this is more likely to have led to an underestimate than overestimate of associations between air pollution and mortality. We cannot rule out an effect of residual confounding on the observed associations but previous work suggests that it is unlikely to have a large impact on our conclusions. The LS does not have information on individual-level smoking, so, as in some other studies,^20^ we used area-level lung cancer risk as a proxy; adjustment for this did not affect observed effect estimates (see online supplementary appendix B table B2). While some comparable studies with individual-level information on smoking have found larger effects in non-smokers,^26^ other studies have found no or only small confounding effects of smoking,^7^ ^9^ ^19^ or that smoking was not related to the exposure,^21^ with larger impacts from adjustments for socioeconomic status.^19^ Association of smoking with air pollution exposures is likely to be through deprivation with which it is highly correlated^33^ and, in the present study, we adjusted for socioeconomic status at both individual and area level.

### Conclusions

This study suggests that air pollution exposure may have persistent long-lasting impacts on mortality risk but that more recent air pollution exposures is associated with higher relative risks than past exposures. Concentration–response function estimates for recent long-term exposures may be slightly overestimated if previous exposures are not taken into account. Findings may be particularly relevant to countries such as China experiencing high but declining levels of particulate concentrations, with a transition from coal to cleaner fuels and increases in emissions from traffic.

## Supplementary Material

Web supplement
