# Supplementary material for: Historic air pollution exposure and long-term mortality risks in England and Wales: prospective longitudinal cohort study
Source: Thorax. 2016 Feb 9;71(4):330–8. doi: 10.1136/thoraxjnl-2015-207111 (PMC4819629; doi:10.1136/thoraxjnl-2015-207111)
Supplement: Web supplement [file thoraxjnl-2015-207111-s1.pdf]

Supplemental information:

Appendix A: General Information
Table A1: International Classification of Disease (ICD) codes used for outcomes:

	ICD-8	ICD-9	ICD-10
All-cause mortality excluding accidents<br>(all codes excluding:)	800 to 999	800 to 999	Chapter V<br>Chapter W<br>Chapter X<br>Y00 to Y98.9
Cardiovascular mortality	393 to 458<br>782	390 to 459<br>671 to 673	Chapter I
Cardiovascular mortality: Coronary heart disease (CHD)	410 to 424	410 to 414.9	I20 to I25.9
Cardiovascular mortality: Stroke	430 to 438	430 to 439.9	I60 to I63.9<br>G46.3<br>G46.4
Respiratory mortality (excludes lung cancer)	460 to 493<br>516<br>518<br>783	460 to 519.9	Chapter J
Respiratory mortality: Respiratory Infections	460 to 466<br>470 to 474<br>480 to 486	460 to 466<br>470 to 478<br>480 to 488	J00 to J06<br>J09 to J18<br>J20 to J22
Respiratory mortality: Chronic Obstructive Pulmonary Disease (COPD)	491 to 492<br>518	490 to 492<br>494<br>496	J40 to J44<br>J47
Lung cancer	162	162	C34, C33

Table A2: Comparison of included and excluded

	Included n=367,658	Excluded n=154,312	X^2
	**Mean(SD)**		
Age in 1971	38 (22.9)	24.7 (19)	**0.000**
**Gender**	**n(%)**	**n(%)**	
Males	175,532 (47.7)	53,428 (54.1)	**0.000**
Females	192,126 (52.3)	45,355 (45.9)	
**Region 1991[1]**			
Northern	18,313 (5.0)	2,063 (2.1)	**0.000**
Yorkshire/Humber	28,462 (7.7)	3,840 (3.9)	
North West	35,086 (9.5)	5,440 (5.5)	
East Midlands	22,791 (6.2)	3,198 (3.2)	
West Midlands	25,146 (6.8)	4,664 (4.7)	
East Anglia	11,605 (3.2)	1,561 (1.6)	
South East	55,335 (15.1)	10,042 (10.1)	
South West	26,248 (7.1)	3,905 (4.0)	
Wales	16,442 (4.5)	2,190 (2.2)	
Inner London	7,742 (2.1)	4,294 (4.4)	
Outer London	18,743 (5.1)	5,845 (5.9)	
Missing region in 1991	*101,742 (27.67)*	*51,739 (52.38)*	
**Individual-level social class 1981[2] (Registrar General occupational categories)**			
I Professional	6,597 (2.12)	1,427 (2.3)	**0.000**
II Intermediate	40,848 (13.1)	7,447 (12.2)	
III(N) Skilled non-manual	44,511 (14.3)	6,834 (11.2)	
III(M) Skilled manual	50,034 (16.0)	8,891 (14.5)	
IV Semi-skilled manual	36,441 (11.7)	7,244 (11.8)	
V Unskilled manual	12,432 (4.0)	2,673 (4.4)	
Armed forces & inadequately described	16,335 (5.0)	635 (1.04)	
Other economically inactive/student/childcare/ Retired/permanently sick	74,793 (24.0)	14,698 (24.0)	
Missing	29,893 (9.6)	11,465 (18.7)	
**Area-level deprivation 1971 (Carstairs quintile)**			
1 (least deprived)	69,608 (19.0)	20,699 (21.0)	**0.000**
2	74,140 (20.2)	18,421 (18.7)	
3	75,428 (20.6)	18,149 (18.4)	
4	75,511 (20.6)	18,315 (18.6)	
5 (most deprived)	72,202 (19.7)	22,945 (23.3)	
**Moved in the 5 year migration 1966-71**			
Movers	144,596 (39.33)	58,236 (59.0)	**0.000**
Non-movers	223,055 (60. 7)	40,547 (41.1)	

[1] Those without region information at 1971 were excluded. [2] Those without social class information at 1971 were excluded. Source: ONS Longitudinal Study (Authors' own work).

Table A3: Air pollution levels in µg/m³ by social class and area-level deprivation and by geographical region

	BS 1971	BS 1981	BS 1991	SO_2 1971	SO_2 1981	SO_2 1991	PM_{10} 2001
	Mean						
Individual social class based on occupation	**1971**	**1981**	**1991**	**1971**	**1981**	**1991**	**2001**
I Professional	39.0	15.7	11.6	81.7	42.6	28.9	20.7
II Intermediate	39.2	15.6	11.6	79.6	42.1	29.0	20.5
III(N) Skilled non-manual	42.5	16.3	11.8	88.8	44.1	29.4	20.7
III(M) Skilled manual	44.5	16.7	12.1	86.8	43.7	30.0	20.6
IV Partly skilled manual	44.1	16.7	12.2	87.0	43.6	30.1	20.6
V Unskilled manual	46.6	16.9	12.1	91.7	44.3	30.1	20.7
Armed forces & inadequately described	41.7	13.2	9.3	85.0	36.8	25.8	20.0
Other economically inactive/student/childcare/Retired or permanently sick	44.1	16.2	11.6	84.4	44	29.3	20.6
Analysis of Variance	**<0.001**	**<0.001**	**<0.001**	**<0.001**	**<0.001**	**<0.001**	**<0.001**
Test for trend (nptrend)	**<0.001**	0.068	**<0.001**	**<0.001**	**<0.001**	**0.010**	**<0.001**
Area-level deprivation (Carstairs quintiles)	**1971**	**1981**	**1991**	**1971**	**1981**	**1991**	**2001**
1 (least deprived)	33.9	14.4	11.3	74.9	39.6	27.7	19.6
2	38.2	14.8	11.0	81.5	39.4	28.1	20.2
3	40.6	15.8	11.4	82.1	41.5	29.2	20.6
4	45.9	17.5	12.4	88.7	45.4	30.9	21.0
5 (most deprived)	54.9	19.0	13.3	99.0	50.6	32.6	22.5
Analysis of Variance	**<0.001**	**<0.001**	**<0.001**	**<0.001**	**<0.001**	**<0.001**	**<0.001**
Test for trend (nptrend)	**<0.001**	**<0.001**	**<0.001**	**<0.001**	**<0.001**	**<0.001**	**<0.001**
Geographical region	**1971**	**1981**	**1991**	**1971**	**1981**	**1991**	**2001**
Northern	65.8	19.2	15.2	89.1	40.6	32.0	20.5
Yorkshire/Humberside	61.6	21.1	16.6	100.4	49.5	36.3	20.3
North West	62.8	19.4	15.6	102.3	45.9	35.2	21.4
East Midlands	47.5	19.5	16.2	84.7	47.0	33.4	19.7
West Midlands	48.9	17.5	13.8	95.9	46.8	32.3	20.0
East Anglia	26.1	13.0	10.3	59.9	37.3	29.6	20.4
South East	24.7	13.4	9.4	62.2	38.6	23.8	20.3
South West	19.0	10.1	4.5	44.1	28.6	21.2	19.6
Wales	22.4	10.2	5.7	44.5	28.2	25.9	12.2
Inner London	45.6	17.0	10.5	150.8	65.3	28.5	27.3
Outer London	37.7	16.6	10.8	115.6	56.3	30.2	24.4
Analysis of Variance	<0.001	<0.001	<0.001	<0.001	<0.001	<0.001	<0.001

Source: ONS Longitudinal Study (Authors' own work)

Appendix B: Supplemental BS and PM_{10} results

Table B1: Logistic regression odds ratios (95% CI) per 10 µg/m^3 for BS exposure in 1981 and 1991 and mortality in subsequent decades shown in main paper in Figure 2

**Decade of outcome**	i) Unadjusted (age and sex only)	ii) Adjusted (age, sex, social class, area-level deprivation, region, pop density)	Sensitivity analysis: Adjusted (age, sex, social class, area-level deprivation, region, pop density & lung cancer)
**Additional outcomes – Exposure in 1971**			
**Cardiovascular mortality: Coronary heart disease (CHD)**			
1972-2009	**1.10 [1.09;1.11]**	**1.03 [1.01;1.05]**	-
1972-1981	**1.11 [1.09;1.13]**	1.03 [0.98;1.08]	-
1982-1991	**1.12 [1.10;1.13]**	**1.04 [1.01;1.08]**	-
1992-2001	**1.09 [1.07;1.10]**	1.03 [1.00;1.06]	-
2002-2009	**1.07 [1.05;1.08]**	1.02 [0.98;1.05]	1.01 [0.99;1.02]
**Cardiovascular mortality: Stroke**			
1972-2009	**1.08 [1.06;1.09]**	**1.05 [1.02;1.09]**	-
1972-1981	**1.12 [1.09;1.16]**	1.05 [0.98;1.13]	-
1982-1991	**1.09 [1.06;1.11]**	1.03 [0.98;1.09]	-
1992-2001	**1.06 [1.04;1.08]**	**1.06 [1.02;1.11]**	-
2002-2009	1.02 [0.99;1.06]	1.03 [0.95;1.12]	1.03 [0.94;1.12]
**Respiratory mortality: Respiratory Infections**			
1972-2009	**1.08 [1.06;1.09]**	**1.06 [1.02;1.11]**	-
1972-1981	**1.10 [1.06;1.15]**	**1.12 [1.01;1.23]**	-
1982-1991	**1.10 [1.06;1.15]**	1.02 [0.92;1.14]	-
1992-2001	**1.08 [1.05;1.10]**	**1.07 [1.02;1.13]**	-
2002-2009	**1.05 [1.03;1.08]**	1.04 [0.98;1.10]	1.02 [0.96;1.08]
**Respiratory mortality: COPD**			
1972-2009	**1.14 [1.12;1.16]**	**1.07 [1.03;1.11]**	-
1972-1981	**1.18 [1.12;1.23]**	**1.12 [1.00;1.25]**	-
1982-1991	**1.16 [1.12;1.20]**	1.02 [0.95;1.10]	-
1992-2001	**1.14 [1.11;1.16]**	**1.09 [1.03;1.15]**	-
2002-2009	**1.12 [1.09;1.14]**	**1.08 [1.02;1.14]**	**1.06 [1.00;1.13]**
**Lung cancer mortality**			
1972-2009	**1.13 [1.12;1.15]**	**1.05 [1.02;1.09]**	-
1972-1981	**1.16 [1.12;1.19]**	**1.08 [1.00;1.16]**	-
1982-1991	**1.15 [1.13;1.18]**	**1.09 [1.04;1.15]**	-
1992-2001	**1.13 [1.10;1.15]**	1.04 [0.99;1.09]	-
2002-2009	**1.11 [1.08;1.13]**	1.04 [0.99;1.09]	-
**Exposure in 1981**			
**All-cause mortality excluding accidents**			
1982-2009	**1.23 [1.20;1.26]**	**1.11 [1.08;1.15]**	**-**
1982-1991	**1.31 [1.26;1.36]**	**1.12 [1.06;1.18]**	-
1992-2001	**1.25 [1.21;1.28]**	**1.13 [1.08;1.18]**	**-**
2002-2009	**1.18 [1.14;1.21]**	**1.09 [1.04;1.13]**	**1.09 [1.04;1.13]**
**Cardiovascular mortality**			
1982-2009	**1.26 [1.22;1.30]**	**1.12 [1.07;1.17]**	-
1982-1991	**1.34 [1.27;1.41]**	**1.13 [1.05;1.22]**	-
1992-2001	**1.28 [1.22;1.33]**	**1.18 [1.11;1.25]**	-
2002-2009	**1.17 [1.12;1.22]**	**1.07 [1.01;1.14]**	1.07 [1.01;1.14]
**Cardiovascular mortality: Coronary heart disease (CHD)**			
1982-2009	**1.32 [1.27;1.36]**	**1.13 [1.08;1.20]**	-
1982-1991	**1.37 [1.29;1.46]**	**1.16 [1.06;1.26]**	-
1992-2001	**1.32 [1.25;1.39]**	**1.17 [1.09;1.26]**	-

2002-2009	**1.22 [1.16;1.29]**	**1.04 [1.01;1.08]**	**1.1 [1.01;1.19]**
**Cardiovascular mortality: Stroke**			
1982-2009	**1.24 [1.16;1.31]**	**1.15 [1.06;1.26]**	-
1982-1991	**1.28 [1.17;1.41]**	1.11 [0.97;1.28]	-
1992-2001	**1.25 [1.16;1.35]**	**1.22 [1.09;1.36]**	-
2002-2009	1.09 [0.95;1.25]	1.09 [0.89;1.33]	1.08 [0.88;1.33]
**Respiratory mortality**			
1982-2009	**1.37 [1.31;1.44]**	**1.20 [1.12;1.28]**	-
1982-1991	**1.52 [1.38;1.68]**	**1.28 [1.10;1.47]**	-
1992-2001	**1.35 [1.26;1.44]**	**1.19 [1.08;1.30]**	-
2002-2009	**1.30 [1.22;1.39]**	**1.18 [1.08;1.29]**	**1.17 [1.06;1.29]**
**Respiratory mortality – Infectious disease**			
1982-2009	**1.25 [1.17;1.34]**	**1.13 [1.03;1.25]**	**-**
1982-1991	**1.36 [1.15;1.61]**	1.14 [0.89;1.46]	**-**
1992-2001	**1.30 [1.19;1.42]**	**1.19 [1.04;1.35]**	**-**
2002-2009	**1.15 [1.05;1.27]**	1.10 [0.96;1.27]	1.08 [0.93;1.24]
**Respiratory mortality - COPD**			
1982-2009	**1.52 [1.42;1.61]**	**1.34 [1.22;1.47]**	**-**
1982-1991	**1.61 [1.42;1.82]**	**1.32 [1.11;1.59]**	**-**
1992-2001	**1.49 [1.35;1.63]**	**1.30 [1.13;1.48]**	**-**
2002-2009	**1.48 [1.34;1.63]**	**1.39 [1.21;1.60]**	**1.41 [1.22;1.62]**
**Lung cancer mortality**			
1982-2009	**1.44 [1.37;1.52]**	**1.16 [1.08;1.25]**	**-**
1982-1991	**1.55 [1.42;1.70]**	**1.21 [1.06;1.38]**	**-**
1992-2001	**1.42 [1.31;1.54]**	**1.15 [1.02;1.29]**	**-**
2002-2009	**1.37 [1.25;1.49]**	1.12 [0.99;1.27]	**-**
**Exposure in 1991**			
**All-cause mortality excluding accidents**			
1992-1991	**1.18 [1.15;1.21]**	**1.13 [1.08;1.18]**	-
1992-2001	**1.22 [1.18;1.26]**	**1.17 [1.11;1.24]**	-
2002-2009	**1.15 [1.11;1.18]**	**1.10 [1.05;1.16]**	**1.10 [1.04;1.16]**
**Cardiovascular mortality**			
1992-1991	**1.22 [1.18;1.27]**	**1.18 [1.11;1.25]**	
1992-2001	**1.29 [1.23;1.35]**	**1.26 [1.16;1.36]**	
2002-2009	**1.15 [1.09;1.10]**	**1.11 [1.03;1.20]**	**1.11 [1.02;1.20]**
**Cardiovascular mortality: Coronary heart disease (CHD)**			
1992-1991	**1.30 [1.25;1.37]**	**1.21 [1.13;1.31]**	-
1992-2001	**1.36 [1.29;1.44]**	**1.29 [1.17;1.42]**	-
2002-2009	**1.22 [1.14;1.30]**	**1.14 [1.03;1.27]**	**1.14 [1.02;1.27]**
**Cardiovascular mortality: Stroke**			
1992-1991	**1.22 [1.13;1.32]**	**1.27 [1.12;1.44]**	-
1992-2001	**1.27 [1.17;1.39]**	**1.32 [1.14;1.53]**	-
2002-2009	1.05 [0.90;1.23]	1.13 [0.88;1.46]	1.10 [0.85;1.43]
**Respiratory mortality**			
1992-1991	**1.29 [1.22;1.36]**	**1.28 [1.16;1.41]**	**-**
1992-2001	**1.31 [1.21;1.41]**	**1.38 [1.22;1.57]**	-
2002-2009	**1.26 [1.17;1.35]**	**1.19 [1.06;1.35]**	**1.17 [1.03;1.33]**
**Respiratory mortality – Infectious disease**			
1992-2009	**1.19 [1.10;1.28]**	**1.19 [1.04;1.36]**	**-**
1992-2001	**1.24 [1.12;1.37]**	**1.31 [1.10;1.55]**	**-**
2002-2009	**1.12 [1.00;1.24]**	1.09 [0.91;1.30]	1.06 [0.88;1.28]
**Respiratory mortality - COPD**			
1992-2009	**1.39 [1.29;1.51]**	**1.44 [1.26;1.65]**	**-**
1992-2001	**1.43 [1.28;1.59]**	**1.53 [1.27;1.83]**	**-**

2002-2009	**1.36 [1.21;1.51]**	**1.36 [1.13;1.64]**	**1.35 [1.11;1.63]**
**Lung cancer mortality**			
1992-2009	**1.38 [1.29;1.48]**	**1.22 [1.09;1.37]**	-
1992-2001	**1.38 [1.26;1.51]**	**1.16 [1.00;1.36]**	-
2002-2009	**1.39 [1.26;1.54]**	**1.31 [1.10;1.55]**	-

Source: ONS Longitudinal Study (Authors' own work)

Table B2: Sensitivity analyses for logistic regression odds ratios (95% CI) per 10 µg/m^3 for BS exposure in 1971 and mortality in subsequent decades

Decade of outcome	i) Unadjusted (age and sex only)	ii) Adjusted (age, sex, social class, area-level deprivation, region, pop density)	Adjusted (age, sex, social class, area-level deprivation, region, pop density & lung cancer)	Adjusted (age, sex, social class, area-level deprivation, region, pop density) - non-movers	Adjusted (age, sex, social class, area-level deprivation, region, pop density and later exposures[1])
**All-cause mortality excluding accidents**					
1972-2009	**1.07 [1.07;1.08]**	**1.03 [1.02;1.05]**		**1.03 [1.01;1.05]**	
1972-1981	**1.10 [1.08;1.11]**	**1.05 [1.02;1.08]**		**1.06 [1.02;1.09]**	
1982-1991	**1.09 [1.08;1.10]**	**1.03 [1.01;1.06]**		**1.03 [1.00;1.06]**	
1992-2001	**1.07 [1.07;1.08]**	**1.04 [1.02;1.06]**		**1.04 [1.02;1.07]**	1.01 [0.99;1.03]
2002-2009	**1.05 [1.05;1.06]**	**1.02 [1.01;1.04]**	**1.02 [1.0;1.04]**	**1.02 [1.00;1.04]**	**1.01 [1.00;1.03]**
**Cardiovascular mortality**					
1972-2009	**1.08 [1.07;1.09]**	**1.03 [1.01;1.05]**		**1.03 [1.01;1.05]**	
1972-1981	**1.11 [1.09;1.13]**	1.03 [0.99;1.08]		1.04 [0.99;1.09]	
1982-1991	**1.10 [1.09;1.12]**	**1.04 [1.01;1.07]**		**1.04 [1.00;1.08]**	
1992-2001	**1.08 [1.07;1.09]**	**1.04 [1.01;1.07]**		**1.04 [1.01;1.08]**	1.00 [0.97;1.03]
2002-2009	**1.05 [1.04;1.06]**	1.01 [0.98;1.04]	1.01 [0.98;1.03]	1.01 [0.98;1.04]	0.99 [0.97;1.03]
**Respiratory mortality**					
1972-2009	**1.11 [1.10;1.12]**	**1.07 [1.04;1.10]**		**1.06 [1.02;1.09]**	
1972-1981	**1.12 [1.09;1.16]**	**1.10 [1.02;1.18]**		**1.14 [1.04;1.24]**	
1982-1991	**1.14 [1.12;1.17]**	1.05 [0.99;1.12]		1.04 [0.97;1.12]	
1992-2001	**1.10 [1.08;1.12]**	**1.08 [1.04;1.13]**		**1.07 [1.02;1.12]**	**1.04 [1.00;1.09]**
2002-2009	**1.09 [1.07;1.11]**	**1.05 [1.01;1.09]**	1.03 [0.99;1.08]	1.03 [0.99;1.08]	1.03 [0.98;1.07]

[1] BS exposure in 1981 and 1991. Source: ONS Longitudinal Study (Authors' own work)

Table B3: Logistic regression odds ratios [95% CI] per 10 µg/m³ for PM_{10} exposure in 2001 and mortality 2002-9 adjusted for past BS and SO_2 exposure

**(2002-2009)**	i) Unadjusted (age and sex only)	ii) Adjusted (age, sex, social class, area-level deprivation, region, pop density)	Sensitivity analysis: Adjusted (age, sex, social class, area-level deprivation, region, pop density & lung cancer)
**All-cause mortality excluding accidents**			
PM_{10} 2001	**1.37 [1.29;1.45]**	**1.24 [1.16;1.34]**	**1.24 [1.15;1.33]**
PM_{10} 2001 + SO_2 1971	**1.24 [1.15;1.32]**	**1.23 [1.15;1.33]**	**1.23 [1.14;1.32]**
PM_{10} 2001 + SO_2 1981	**1.22 [1.14;1.31]**	**1.17 [1.08;1.26]**	**1.16 [1.08;1.26]**
PM_{10} 2001 + SO_2 1991	**1.25 [1.17;1.33]**	**1.18 [1.09;1.27]**	**1.17 [1.09;1.27]**
PM_{10} 2001 + SO_2 all years	**1.19 [1.11;1.29]**	**1.14 [1.06;1.24]**	**1.14 [1.05;1.23]**
PM_{10} 2001 + BS 1971 + SO_2 1971	**1.32 [1.23;1.41]**	**1.24 [1.15;1.33]**	**1.23 [1.15;1.33]**
PM_{10} 2001 + BS 1981 + SO_2 1981	**1.30 [1.21;1.40]**	**1.18 [1.09;1.27]**	**1.18 [1.09;1.27]**
PM_{10} 2001 + BS 1991 + SO_2 1991	**1.25 [1.18;1.34]**	**1.19 [1.11;1.29]**	**1.19 [1.10;1.28]**
PM_{10} 2001 + BS and SO_2 all years	**1.23 [1.14;1.33]**	**1.16 [1.07;1.26]**	**1.16 [1.07;1.26]**
**Cardiovascular mortality**			
PM_{10} 2001	**1.25 [1.14;1.37]**	**1.13 [1.01;1.25]**	**1.12 [1.01;1.25]**
PM_{10} 2001 + SO_2 1971	**1.14 [1.03;1.26]**	1.12 [0.99;1.26]	1.12 [1.00;1.25]
PM_{10} 2001 + SO_2 1981	**1.15 [1.03;1.27]**	1.08 [0.96;1.21]	1.08 [0.96;1.21]
PM_{10} 2001 + SO_2 1991	**1.14 [1.03;1.25]**	1.07 [0.96;1.22]	1.07 [0.95;1.20]
PM_{10} 2001 + SO_2 all years	1.11 [0.99;1.24]	1.05 [0.93;1.18]	1.05 [0.93;1.18]
PM_{10} 2001 + BS 1971 + SO_2 1971	**1.22 [1.10;1.36]**	1.13 [1.01;1.26]	**1.12 [1.01;1.26]**
PM_{10} 2001 + BS 1981 + SO_2 1981	**1.24 [1.11;1.38]**	1.10 [0.97;1.23]	1.09 [0.97;1.23]
PM_{10} 2001 + BS 1991 + SO_2 1991	**1.15 [1.04;1.26]**	1.10 [0.98;1.23]	1.09 [0.98;1.23]
PM_{10} 2001 + BS and SO_2 all years	**1.16 [1.03;1.30]**	1.08 [0.95;1.22]	1.07 [0.95;1.21]
**Respiratory mortality**			
PM_{10} 2001	**1.55 [1.35;1.77]**	**1.23 [1.05;1.45]**	**1.22 [1.04;1.44]**
PM_{10} 2001 + SO_2 1971	**1.26 [1.08;1.47]**	**1.22 [1.03;1.44]**	**1.21 [1.02;1.43]**
PM_{10} 2001 + SO_2 1981	**1.30 [1.11;1.53]**	1.15 [0.96;1.37]	1.14 [0.96;1.36]
PM_{10} 2001 + SO_2 1991	**1.41 [1.22;1.62]**	1.16 [0.98;1.37]	1.15 [0.97;1.36]
PM_{10} 2001 + SO_2 all years	**1.30 [1.09;1.54]**	1.15 [0.96;1.38]	1.14 [0.95;1.37]
PM_{10} 2001 + BS 1971 + SO_2 1971	**1.40 [1.19;1.65]**	**1.24 [1.05;1.47]**	**1.23 [1.04;1.46]**
PM_{10} 2001 + BS 1981 + SO_2 1981	**1.45 [1.23;1.71]**	1.18 [0.99;1.41]	1.17 [0.98;1.40]
PM_{10} 2001 + BS 1991 + SO_2 1991	**1.40 [1.21;1.62]**	1.17 [0.98;1.38]	1.15 [0.97;1.37]
PM_{10} 2001 + BS and SO_2 all years	**1.34 [1.12;1.59]**	1.19 [0.99;1.43]	1.18 [0.98;1.42]

Source: ONS Longitudinal Study (Authors' own work)

Table B4: Logistic regression odds ratios [95% CI] per 10 µg/m^3 for PM_{10} exposure in 2001 and subgroup analyses of mortality 2002-9

**(2002-2009)**	i) Unadjusted (age and sex only)	ii) Adjusted (age, sex, social class, area-level deprivation, region, pop density)	Sensitivity analysis: Adjusted (age, sex, social class, area-level deprivation, region, pop density & lung cancer)
**All-cause mortality excluding accidents**			
PM_{10} 2001	**1.37 [1.29;1.45]**	**1.24 [1.16;1.34]**	**1.24 [1.15;1.33]**
**Cardiovascular mortality**			
PM_{10} 2001	**1.25 [1.14;1.37]**	**1.13 [1.01;1.25]**	**1.12 [1.01;1.25]**
**Cardiovascular mortality: Coronary heart disease (CHD)**			
PM_{10} 2001	**1.36 [1.21;1.53]**	**1.21 [1.05;1.4]**	**1.21 [1.05;1.39]**
**Cardiovascular mortality: Stroke**			
PM_{10} 2001	0.85 [0.63;1.15]	0.86 [0.60;1.22]	0.84 [0.59;1.20]
**Respiratory mortality**			
PM_{10} 2001	**1.55 [1.35;1.77]**	**1.23 [1.05;1.45]**	**1.22 [1.04;1.44]**
**Respiratory mortality: Infectious disease**			
PM_{10} 2001	**1.35 [1.11;1.64]**	1.17 [0.92;1.49]	**1.16 [0.91;1.47]**
**Respiratory mortality: COPD**			
PM_{10} 2001	**1.83 [1.49;2.24]**	**1.32 [1.04;1.68]**	**1.31 [1.03;1.68]**
**Lung cancer**			
PM_{10} 2001	**1.98 [1.65;2.38]**	**1.60 [1.29;1.99]**	**-**

Source: ONS Longitudinal Study (Authors' own work)

Figure B1: Logistic regression odds ratios[1] [95% CI] per 10 µg/m³ for PM_{10} exposure in 2001 and mortality 2002-9 stratified by highest and lowest BS tertiles

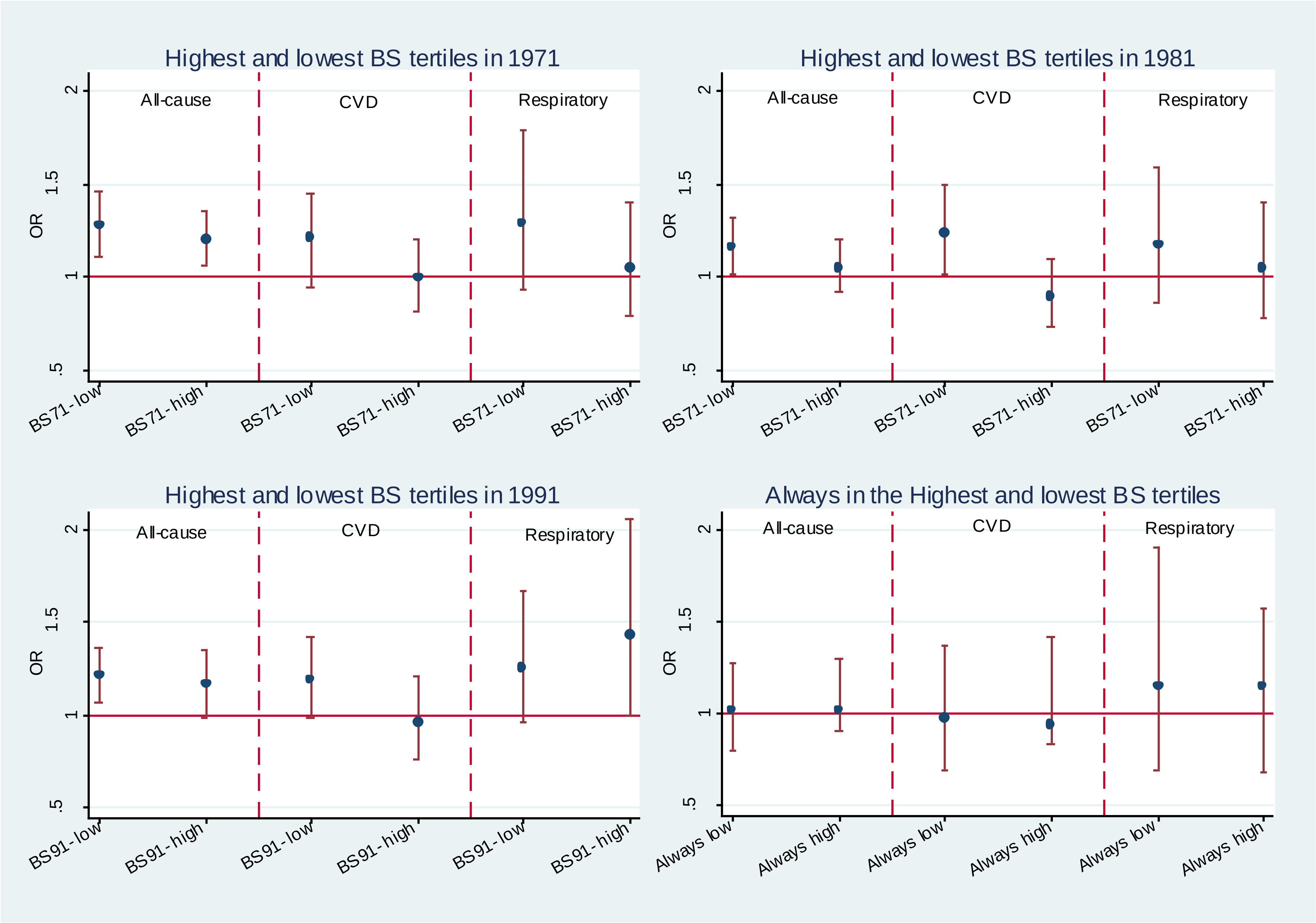

[1]Adjusted for sex, age, social class, area-level deprivation, region & population density. Source: ONS Longitudinal Study (Authors' own work)

Appendix C: SO_2 Results

Figure C1: Logistic regression odds ratios (95% CI) per 10 µg/m^3 for SO_2 exposure in 1971, 1981 and 1991 and mortality in subsequent decades[1]

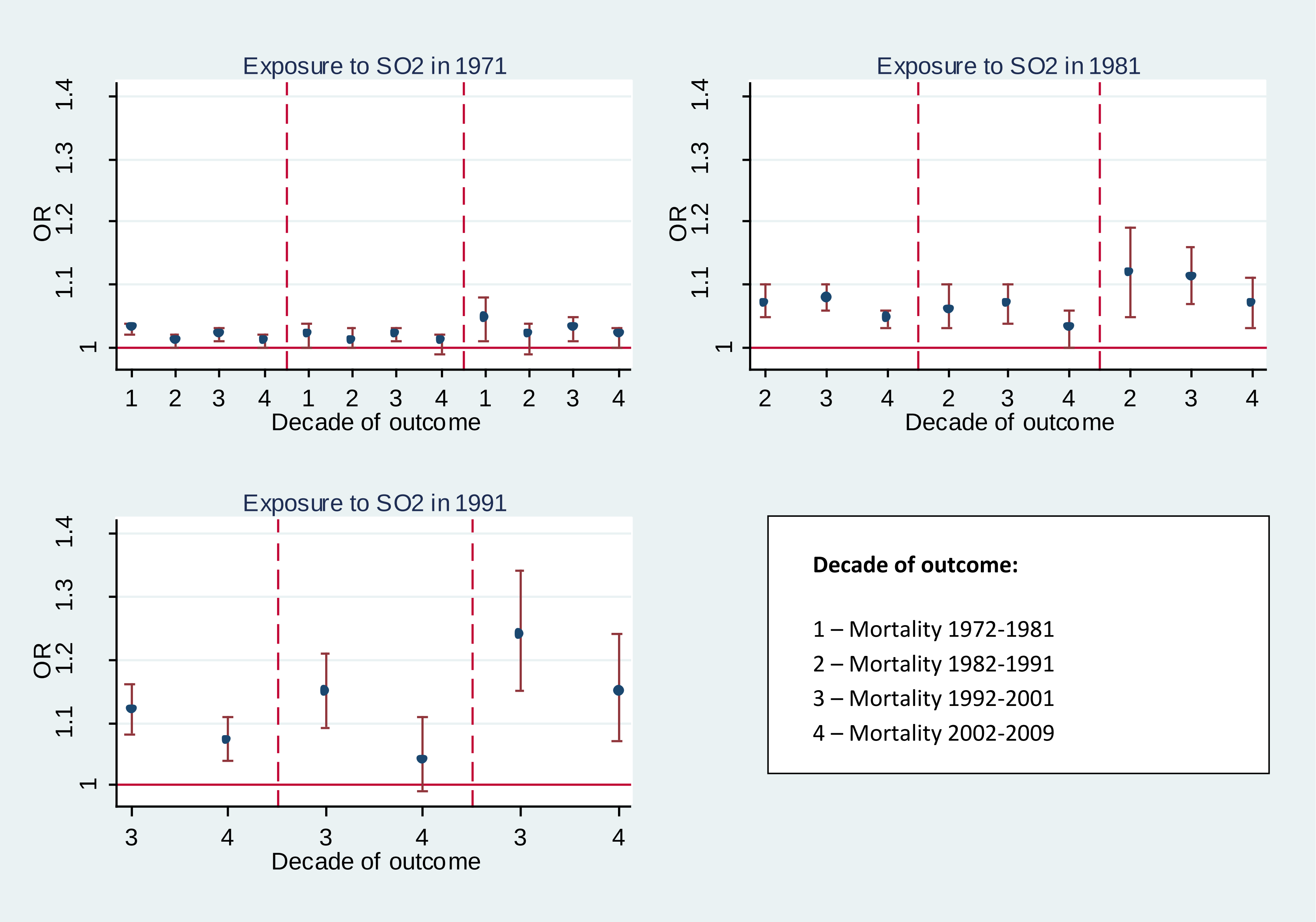

Decade of outcome:

1 – Mortality 1972-1981
2 – Mortality 1982-1991
3 – Mortality 1992-2001
4 – Mortality 2002-2009

[1]Adjusted for for age and sex, social class of individual and area, population density and geographical region. Source: ONS Longitudinal Study (Authors' own work)

Table C1: Logistic regression odds ratios (95% CI) per 10 μg/m^3 for SO_2 exposure in 1971, 1981 and 1991 and mortality in subsequent decades.

Decade of outcome	i) Unadjusted (age and sex only)	ii) Adjusted (age, sex, social class, area-level deprivation, region, pop density)	Sensitivity analysis: Adjusted (age, sex, social class, area-level deprivation, region, pop density & lung cancer)
	**Exposure in 1971**		
**All-cause mortality excluding accidents**			
1972-2009	**1.04 [1.03;1.04]**	**1.02 [1.01;1.02]**	-
1972-1981	**1.04 [1.03;1.05]**	**1.03 [1.02;1.04]**	-
1982-1991	**1.04 [1.03;1.05]**	**1.01 [1.01;1.02]**	-
1992-2001	**1.04 [1.03;1.04]**	**1.02 [1.01;1.03]**	-
2002-2009	**1.02 [1.02;1.03]**	**1.01 [1.01;1.02]**	**1.01 [1.00;1.02]**
**Cardiovascular mortality**			
1972-2009	**1.03 [1.03;1.04]**	**1.01 [1.01;1.02]**	-
1972-1981	**1.04 [1.03;1.05]**	**1.02 [1.00;1.04]**	-
1982-1991	**1.03 [1.03;1.04]**	**1.01 [1.00;1.03]**	-
1992-2001	**1.03 [1.02;1.04]**	**1.02 [1.01;1.03]**	-
2002-2009	**1.02 [1.01;1.03]**	1.01 [0.99;1.02]	1.00 [0.99;1.01]
**Cardiovascular mortality: Coronary heart disease (CHD)**			
1972-2009	**1.03 [1.03;1.04]**	**1.02 [1.01;1.03]**	-
1972-1981	**1.03 [1.02;1.05]**	**1.02 [1.01;1.04]**	-
1982-1991	**1.03 [1.02;1.04]**	1.01 [1.00;1.03]	-
1992-2001	**1.03 [1.03;1.04]**	**1.02 [1.01;1.03]**	-
2002-2009	**1.03 [1.02;1.03]**	1.01 [0.99;1.02]	1.01 [0.99;1.02]
**Cardiovascular mortality: Stroke**			
1972-2009	**1.02 [1.01;1.03]**	1.02 [1.00;1.03]	-
1972-1981	**1.03 [1.01;1.04]**	1.01 [0.98;1.04]	-
1982-1991	**1.02 [1.01;1.04]**	1.00 [0.98;1.03]	-
1992-2001	**1.02 [1.01;1.03]**	1.02 [1.00;1.04]	-
2002-2009	1.00 [0.98;1.02]	1.02 [0.98;1.06]	1.01 [0.97;1.04]
**Respiratory mortality**			
1972-2009	**1.05 [1.04;1.06]**	**1.03 [1.01;1.04]**	-
1972-1981	**1.05 [1.03;1.07]**	**1.05 [1.01;1.08]**	-
1982-1991	**1.06 [1.04;1.07]**	1.02 [0.99;1.04]	-
1992-2001	**1.05 [1.04;1.06]**	**1.03 [1.01;1.05]**	-
2002-2009	**1.04 [1.03;1.05]**	**1.02 [1.00;1.03]**	1.01 [0.99;1.03]
**Respiratory mortality: Respiratory Infections**			
1972-2009	**1.04 [1.03;1.05]**	**1.03 [1.01;1.05]**	
1972-1981	**1.05 [1.03;1.07]**	**1.06 [1.02;1.11]**	
1982-1991	**1.04 [1.02;1.07]**	1.01 [0.97;1.06]	
1992-2001	**1.05 [1.03;1.06]**	**1.03 [1.01;1.06]**	
2002-2009	**1.03 [1.01;1.04]**	1.02 [0.99;1.04]	1.01 [0.98;1.03]
**Respiratory mortality: COPD**			
1972-2009	**1.06 [1.05;1.07]**	**1.03 [1.01;1.04]**	
1972-1981	**1.06 [1.03;1.08]**	1.04 [0.99;1.09]	
1982-1991	**1.06 [1.04;1.08]**	1.01 [0.98;1.04]	
1992-2001	**1.06 [1.04;1.07]**	**1.03 [1.01;1.05]**	
2002-2009	**1.06 [1.04;1.07]**	**1.03 [1.01;1.05]**	**1.02 [1.00;1.05]**
**Lung cancer mortality**			
1972-2009	**1.07 [1.06;1.07]**	**1.04 [1.02;1.05]**	
1972-1981	**1.07 [1.05;1.09]**	**1.04 [1.01;1.07]**	
1982-1991	**1.08 [1.06;1.09]**	**1.05 [1.03;1.07]**	
1992-2001	**1.06 [1.05;1.07]**	**1.03 [1.01;1.05]**	
2002-2009	**1.05 [1.04;1.07]**	**1.03 [1.01;1.05]**	

	**Exposure in 1981**		
**All-cause mortality excluding accidents**			
1982-2009	**1.09 [1.08;1.10]**	**1.07 [1.05;1.08]**	-
1982-1991	**1.10 [1.09;1.12]**	**1.07 [1.05;1.10]**	-
1992-2001	**1.10 [1.08;1.11]**	**1.08 [1.06;1.10]**	-
2002-2009	**1.07 [1.05;1.08]**	**1.05 [1.03;1.06]**	**1.04 [1.02;1.06]**
**Cardiovascular mortality**			
1982-2009	**1.08 [1.06;1.09]**	**1.06 [1.04;1.08]**	-
1982-1991	**1.08 [1.06;1.10]**	**1.06 [1.03;1.10]**	-
1992-2001	**1.08 [1.06;1.10]**	**1.07 [1.04;1.10]**	-
2002-2009	**1.05 [1.03;1.07]**	**1.03 [1.00;1.06]**	1.03 [1.00;1.05]
**Cardiovascular mortality: Coronary heart disease (CHD)**			
1982-2009	**1.08 [1.07;1.10]**	**1.06 [1.04;1.09]**	-
1982-1991	**1.08 [1.05;1.11]**	**1.07 [1.03;1.11]**	-
1992-2001	**1.08 [1.06;1.11]**	**1.07 [1.04;1.10]**	-
2002-2009	**1.07 [1.04;1.09]**	**1.04 [1.01;1.08]**	1.04 [1.00;1.08]
**Cardiovascular mortality: Stroke**			
1982-2009	**1.05 [1.02;1.07]**	**1.04 [1.01;1.08]**	-
1982-1991	1.05 [1.00;1.09]	1.01 [0.96;1.07]	-
1992-2001	**1.06 [1.02;1.09]**	**1.06 [1.01;1.11]**	-
2002-2009	1.00 [0.95;1.07]	1.04 [0.96;1.13]	1.02 [0.94;1.11]
**Respiratory mortality**			
1982-2009	**1.14 [1.11;1.15]**	**1.10 [1.07;1.13]**	-
1982-1991	**1.16 [1.11;1.21]**	**1.12 [1.05;1.19]**	-
1992-2001	**1.14 [1.11;1.17]**	**1.11 [1.07;1.16]**	-
2002-2009	**1.10 [1.07;1.13]**	**1.07 [1.03;1.11]**	**1.06 [1.02;1.10]**
**Respiratory mortality: Respiratory Infections**			
1982-2009	**1.12 [1.09;1.15]**	**1.09 [1.04;1.13]**	-
1982-1991	**1.14 [1.06;1.22]**	1.10 [1.00;1.22]	-
1992-2001	**1.15 [1.11;1.19]**	**1.12 [1.07;1.18]**	-
2002-2009	**1.05 [1.01;1.10]**	1.04 [0.99;1.11]	1.03 [0.97;1.09]
**Respiratory mortality: COPD**			
1982-2009	**1.16 [1.13;1.19]**	**1.14 [1.09;1.18]**	-
1982-1991	**1.17 [1.12;1.24]**	**1.12 [1.04;1.21]**	-
1992-2001	**1.15 [1.11;1.20]**	**1.13 [1.07;1.19]**	-
2002-2009	**1.16 [1.12;1.21]**	**1.14 [1.08;1.21]**	**1.14 [1.07;1.21]**
**Lung cancer mortality**			
1982-2009	**1.17 [1.14;1.19]**	**1.11 [1.08;1.15]**	-
1982-1991	**1.20 [1.15;1.25]**	**1.13 [1.07;1.19]**	-
1992-2001	**1.14 [1.10;1.18]**	**1.10 [1.04;1.15]**	-
2002-2009	**1.14 [1.10;1.19]**	**1.11 [1.05;1.17]**	-
	**Exposure in 1991**		
**All-cause mortality excluding accidents**			
1992-2009	**1.16 [1.14;1.19]**	**1.10 [1.07;1.13]**	-
1992-2001	**1.20 [1.17;1.23]**	**1.12 [1.08;1.16]**	-
2002-2009	**1.13 [1.10;1.16]**	**1.07 [1.04;1.11]**	**1.07 [1.03;1.11]**
**Cardiovascular mortality**			
1992-2009	**1.18 [1.15;1.21]**	**1.09 [1.05;1.14]**	-
1992-2001	**1.23 [1.19;1.27]**	**1.15 [1.09;1.21]**	-
2002-2009	**1.12 [1.08;1.16]**	1.04 [0.99;1.10]	1.04 [0.99;1.09]
**Cardiovascular mortality: Coronary heart disease (CHD)**			
1992-1991	**1.24 [1.20;1.28]**	**1.13 [1.07;1.18]**	-
1992-2001	**1.28 [1.23;1.33]**	**1.17 [1.11;1.25]**	-
2002-2009	**1.18 [1.13;1.23]**	1.07 [1.00;1.15]	1.07 [1.00;1.15]

**Cardiovascular mortality: Stroke**			
1992-1991	**1.15 [1.09;1.22]**	**1.11 [1.03;1.21]**	-
1992-2001	**1.18 [1.11;1.26]**	**1.14 [1.04;1.25]**	-
2002-2009	1.05 [0.94;1.17]	1.04 [0.88;1.22]	1.03 [0.87;1.22]
**Respiratory mortality**			
1992-2009	**1.26 [1.21;1.31]**	**1.19 [1.12;1.26]**	-
1992-2001	**1.28 [1.21;1.35]**	**1.24 [1.15;1.34]**	-
2002-2009	**1.23 [1.17;1.30]**	**1.15 [1.07;1.24]**	**1.14 [1.05;1.23]**
**Respiratory mortality: Respiratory Infections**			
1992-2009	**1.17 [1.11;1.24]**	**1.13 [1.04;1.23]**	-
1992-2001	**1.22 [1.14;1.31]**	**1.22 [1.10;1.36]**	-
2002-2009	**1.11 [1.03;1.20]**	1.05 [0.94;1.17]	1.03 [0.92;1.16]
**Respiratory mortality: COPD**			
1992-2009	**1.36 [1.29;1.44]**	**1.31 [1.20;1.42]**	-
1992-2001	**1.37 [1.28;1.48]**	**1.30 [1.16;1.45]**	-
2002-2009	**1.34 [1.24;1.45]**	**1.31 [1.17;1.47]**	**1.31 [1.16;1.47]**
**Lung cancer mortality**			
1992-2009	**1.31 [1.25;1.37]**	**1.18 [1.10;1.27]**	-
1992-2001	**1.32 [1.24;1.41]**	**1.16 [1.05;1.27]**	-
2002-2009	**1.30 [1.21;1.40]**	**1.21 [1.09;1.34]**	-

Source: ONS Longitudinal Study (Authors' own work)

Figure C2: Logistic regression odds ratios (95% CI) per 10 μg/m^3 for SO_2 exposure in 1971, 1981 and 1991 and mortality in each subsequent decade[1]

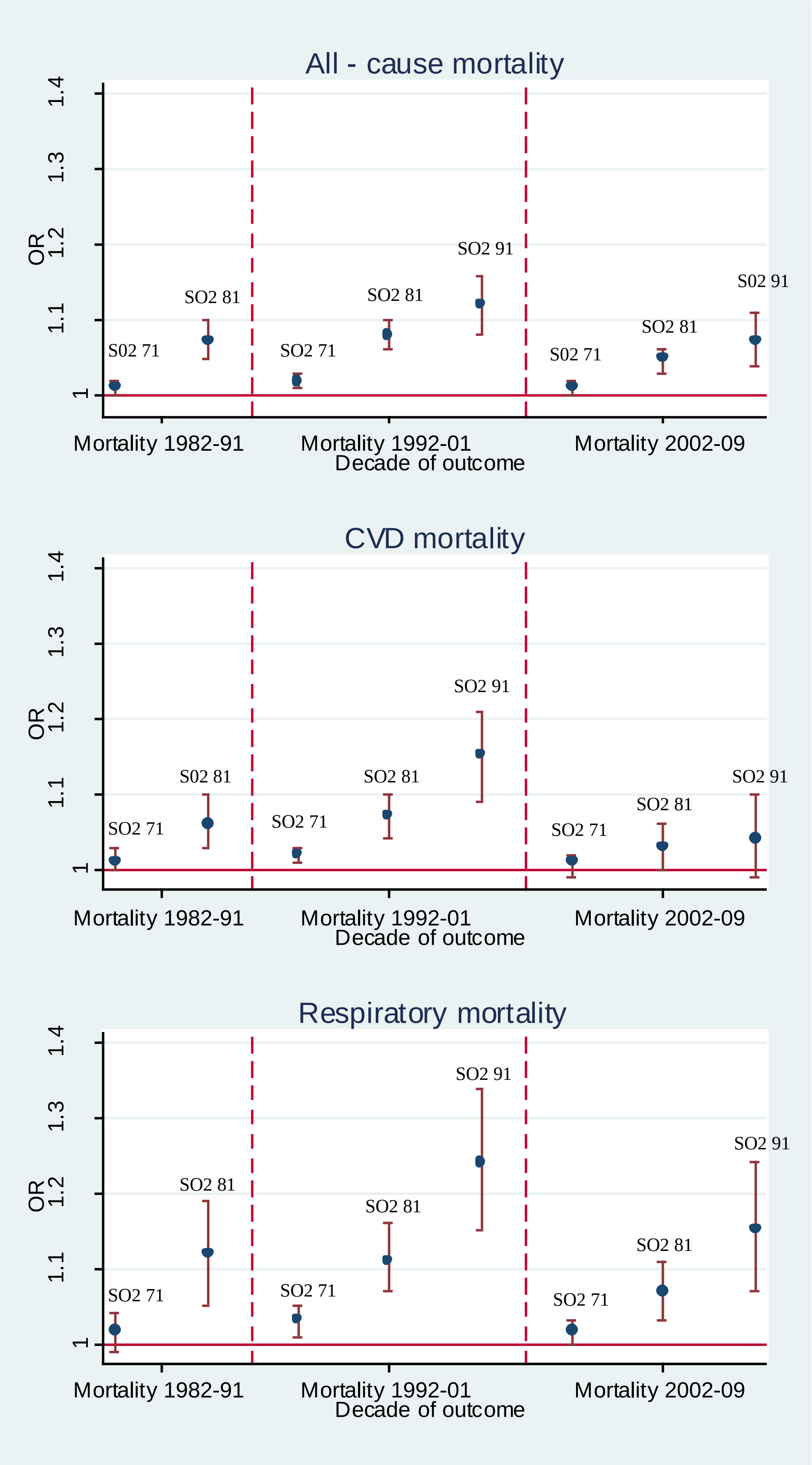

[1]Adjusted for for age and sex, social class of individual and area, population density and geographical region. Source: ONS Longitudinal Study (Authors' own work)

Figure C3: Logistic regression odds ratios[1] [95% CI] per 10 μg/m³ for PM_{10} exposure in 2001 and mortality 2002-9 stratified by highest and lowest SO_2 tertiles

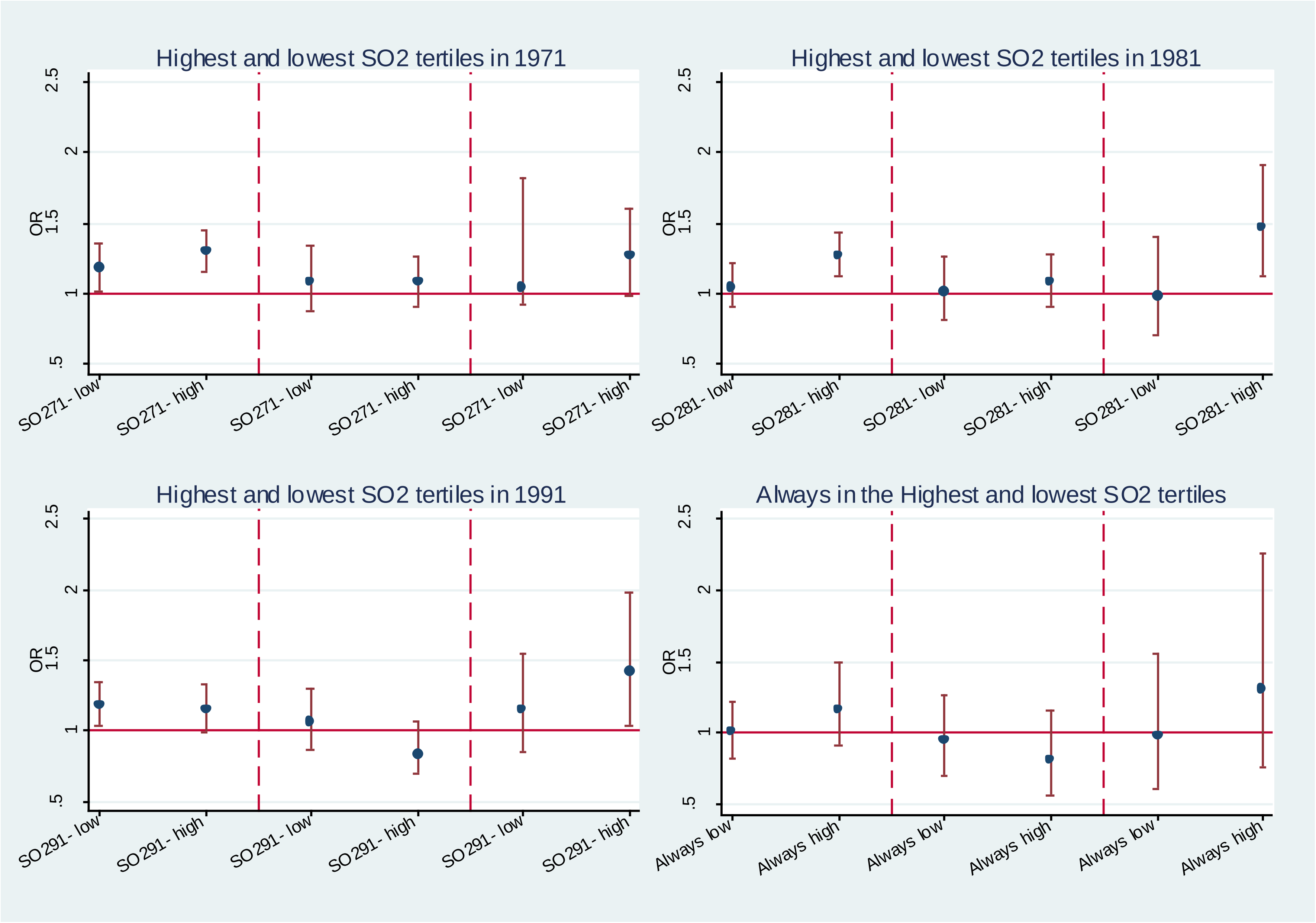

[1]Adjusted for sex, age, social class, area-level deprivation, region, & population density. Source: ONS Longitudinal Study (Authors' own work)

Figure C4: Concentration-response for tertiles of SO_2 exposure in1971 and subsequent mortality 1971 – 2009 for all-cause, CVD and respiratory mortality

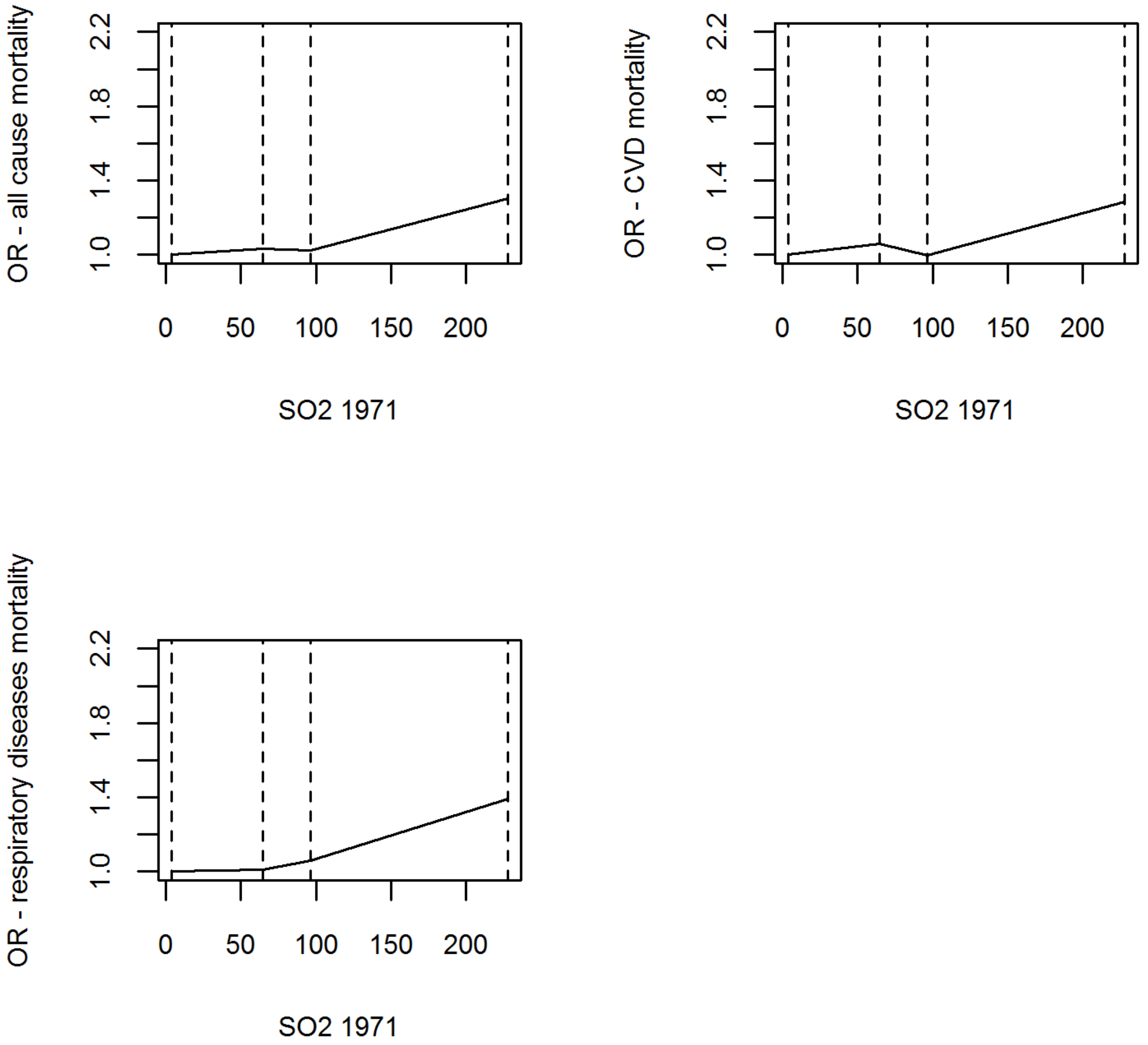

[1]Adjusted for sex, age, social class, area-level deprivation, region & population density. Source: ONS Longitudinal Study (Authors' own work)
